# Cerebellar Continuous Theta Burst Stimulation for Insomnia Disorder: A Randomized Sham‐Controlled Trial

**DOI:** 10.1002/cns.71231

**Published:** 2026-10-26

**Authors:** Xin‐Yao Liu, Dong Dong, Chang‐Geng Song, Yu‐Xuan Ma, Dian‐Wei Wu, Xuan Wang, Jian‐Min Hao, Hui Li, Jing‐Jing Zhao, Geng‐Yao Hu, Feng Xia, Wen Jiang

**Affiliations:** ^1^ Department of Neurology, Xijing Hospital Fourth Military Medical University Xi'an China

**Keywords:** brain network, cerebellum, continuous theta burst stimulation, insomnia disorder, transcranial magnetic stimulation

## Abstract

**Objective:**

To evaluate the effects of cerebellar continuous theta burst stimulation (cTBS) in insomnia disorder (ID).

**Methods:**

In this randomized sham‐controlled trial, patients with ID received 2 weeks of active or sham cerebellar cTBS. The primary outcome was a ≥ 3‐point improvement in Pittsburgh Sleep Quality Index (PSQI) score after treatment. Secondary outcomes included changes in sleep, mood, and resting‐state fMRI.

**Results:**

Seventy‐two patients (51 women; mean age, 44.3 ± 11.6 years) were included in the intention‐to‐treat analysis. More patients in the active than sham group achieved a ≥ 3‐point PSQI improvement after treatment (75.0% vs. 33.3%; adjusted OR, 6.06; 95% CI, 2.17–18.50; *p* < 0.001). Active cTBS reduced PSQI scores versus sham both post‐treatment (adjusted mean difference, −3.34; 95% CI, −4.57 to −2.12; *p* < 0.001) and at 1 month (−3.70; 95% CI, −4.93 to −2.48; *p* < 0.001), and also improved Insomnia Severity Index, Hamilton Anxiety Scale, and Hamilton Depression Scale at both time points (all *p* < 0.01). Exploratory fMRI analyses identified several post‐treatment connectivity differences between groups, but only right amygdala–right orbitofrontal connectivity showed a significant group‐by‐time interaction (FDR‐adjusted *p* = 0.038). No serious adverse events occurred.

**Conclusion:**

Cerebellar cTBS was well tolerated and promising for ID.

**Trial Registration:**

ClinicalTrials.gov (NCT05992285)

## Graphical abstract

Liu et al. show that cerebellar continuous theta burst stimulation is associated with improved clinical symptoms in patients with insomnia disorder in a randomized sham‐controlled trial. Exploratory fMRI analyses suggest associated changes in distributed sleep‐related brain networks, supporting further investigation of the cerebellum as a neuromodulation target for insomnia.

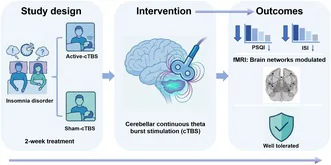

## 1 Introduction

Insomnia disorder (ID), characterized by persistent difficulty initiating or maintaining sleep and by non‐restorative sleep [1], is highly prevalent and affects up to 50% of patients in primary care settings [2]. It substantially impairs quality of life and is associated with increased risks of cardiovascular disease, metabolic disorders, immune dysfunction, neuropsychiatric conditions, and accidental injury, creating a considerable socioeconomic burden [2, 3]. Although cognitive behavioral therapy for insomnia is recommended as first‐line treatment, its use in clinical practice is often limited by poor accessibility, suboptimal adherence, and delayed therapeutic effects [4]. Pharmacological treatments may provide faster symptom relief but are constrained by risks of dependence, relapse, and cognitive adverse effects [5]. Together, these limitations highlight the need for safe, effective, and accessible alternative treatments for ID.

Transcranial magnetic stimulation (TMS) is a non‐invasive neuromodulation technique that uses rapidly changing magnetic fields to alter cortical excitability [6, 7]. Increasing evidence suggests that TMS is a promising treatment for insomnia, with potential advantages over pharmacotherapy in improving sleep quality and sustaining therapeutic benefit [8, 9]. Although traditionally linked to motor control, the cerebellum is now recognized as an important regulator of higher‐order brain functions [10, 11]. We previously showed that cerebellar TMS can be therapeutically effective in drug‐resistant epilepsy [12] and disorders of consciousness [13], highlighting the cerebellum's potential as a therapeutic TMS target. While classical models of sleep regulation focus on the brainstem, hypothalamus, thalamus, and cortex, growing evidence suggests that the cerebellum also contributes to sleep onset, maintenance, and microstructure via its connectivity with arousal and thalamo‐cortical systems, together with circadian and genetic influences [14, 15, 16, 17]. In insomnia, increased connectivity between cerebellar subregions (e.g., Crus I/II and lobules VI–VIII) and arousal networks suggests that the cerebellum may contribute to hyperarousal and impaired sensory filtering [18, 19, 20, 21]. Classic TMS for insomnia primarily targets the dorsolateral prefrontal cortex (DLPFC). Although it can improve sleep and mood symptoms, its effects are largely mediated by the cognitive‐emotional control network and may not fully address the core mechanisms of hyperarousal and autonomic imbalance [22]. In contrast, the cerebellum is extensively connected to the brainstem, thalamus, limbic system, and cortex, allowing it to influence sleep regulation more broadly through the dentate‐thalamo‐cortical and cerebellar‐limbic pathways [17, 23]. Therefore, as a novel neuromodulation target for insomnia, we hypothesized that the cerebellum may offer stronger network integration and a more pathophysiologically plausible basis than conventional prefrontal stimulation. Compared with conventional low‐frequency repetitive transcranial magnetic stimulation (rTMS) or intermittent theta burst stimulation (TBS), continuous TBS (cTBS) may be a more relevant and efficient approach because it more strongly induces neuroplasticity, enhances GABAergic inhibition, and promotes slow‐wave activity [24].

In this randomized, sham‐controlled trial, we evaluated the efficacy and safety of cerebellar cTBS in patients with ID. As a TMS protocol, cTBS can induce lasting neuromodulatory effects with substantially shorter treatment time, which may improve feasibility, adherence, and cost‐effectiveness [25, 26]. To explore potential mechanisms, we also used functional MRI (fMRI) to assess treatment‐related changes in brain network connectivity. We hypothesized that cerebellar cTBS would improve insomnia symptoms by modulating functional connectivity within brain networks involved in sleep regulation.

## 2 Methods

### 2.1 Study Design

This single‐center, randomized, sham‐controlled trial was conducted at the Department of Neurology, Xijing Hospital, Fourth Military Medical University, Shaanxi, China. The complete study protocol is available in the File S1. All participants (or their parent/legal guardian/next of kin) provided written informed consent.

### 2.2 Patients

Participants were recruited from 16 August 2023 to 29 September 2024. Inclusion criteria were: (1) age 18–60 years; (2) Diagnostic and Statistical Manual of Mental Disorders, Fifth Edition (DSM‐5) diagnosis of ID, Pittsburgh Sleep Quality Index (PSQI) score > 10, Hamilton Anxiety Scale (HAMA) score ≤ 14, and Hamilton Depression Scale (HAMD) score ≤ 17 [27, 28], to exclude individuals with moderate‐to‐severe anxiety or depression, so that the study could focus more specifically on the effects of the intervention on insomnia; (3) no use of medications or supplements affecting sleep within 1 month before enrollment; and (4) ability to comply with the study protocol and complete all assessments. Exclusion criteria were: (1) comorbid sleep disorders, epilepsy, psychiatric, endocrine, or other neurological disorders (e.g., Parkinson's disease or Alzheimer's disease); (2) transmeridian travel or night shift work within the previous month; and (3) contraindications to TMS or MRI (File S3, Table S1).

### 2.3 Randomization and Masking

Participants were randomized 1:1 to Active‐cTBS or Sham‐cTBS using a computer‐generated schedule (SAS 9.2). Allocation was generated and maintained by an independent unmasked statistician. Participants and investigators involved in recruitment, assessment, and data analysis were blinded to group assignment. The TMS operator accessed allocation only to administer the assigned intervention and was not involved in outcome assessment or data analysis. Outcomes were assessed by a single trained blinded assessor using a standardized procedure; thus, inter‐rater reliability was not applicable. Detailed methods are described in the File S2.

### 2.4 Procedures

The trial consisted of a 2‐week treatment period and a 4‐week follow‐up. Before treatment, all patients underwent clinical assessments, including the PSQI, Epworth Sleepiness Scale (ESS), Insomnia Severity Index (ISI), HAMD, and HAMA, together with fMRI. The same clinical assessments and fMRI were repeated immediately after treatment to evaluate immediate effects, and clinical scales were reassessed 1 month after treatment to evaluate longer‐term effects.

Patients received active or sham stimulation once daily on weekdays for 2 weeks (10 sessions), all during daytime hours. Stimulation was delivered using a Neuro‐MS/D stimulator (Neurosoft, GTMEDI Technology Co.) with a double‐cone coil (external loop diameter, 125 mm; peak magnetic field, 4 T) positioned tangentially over the occipital scalp. Individual MRI‐guided frameless neuronavigation (Brain Science Tools BV, Utrecht, the Netherlands) was used to target cerebellar lobule VIIb by focusing on the cerebellar dentate nucleus (CDN). Active cTBS consisted of bursts of 3 pulses at 50 Hz repeated at 5 Hz (600 pulses/40 s). The right and left cerebellar hemispheres were stimulated sequentially twice with 5‐min intervals (right‐left‐right‐left), yielding 1200 pulses per side. Stimulation intensity was set at 80% resting motor threshold (RMT), determined at treatment initiation as the minimum intensity evoking abductor pollicis brevis responses in at least 5 of 10 trials during single‐pulse stimulation of the primary motor cortex for the dominant thumb area, with participants seated and muscles relaxed. Sham stimulation used identical targeting and stimulation parameters, but the coil was rotated 90° and contacted only at the edge to minimize effective stimulation while preserving acoustic and somatosensory sensations. Posture, localization, session duration, and operator instructions were identical between groups to maintain blinding [29, 30, 31, 32, 33]. Participants remained seated and wore in‐ear noise‐canceling headphones throughout treatment.

### 2.5 Outcomes

The primary outcome was the proportion of patients with a ≥ 3‐point improvement in PSQI score after 2 weeks of treatment (10 stimulation sessions). The PSQI is a widely used and validated measure of global sleep quality, and a ≥ 3‐point reduction was selected because it has been regarded in previous studies as a clinically meaningful improvement [34, 35]. Secondary efficacy outcomes included (1) differences in PSQI, ESS, ISI, HAMA, and HAMD scores assessed both post‐treatment and at the 1‐month follow‐up; (2) between‐group differences in fMRI measures following 2 weeks of treatment. Safety was assessed by the incidence of adverse events (AEs) and serious adverse events (SAEs) throughout the study, which were solicited and recorded after every treatment session and during follow‐up assessments. Common cTBS‐related AEs included dizziness, headache, tinnitus, and scalp discomfort; in case of SAE (e.g., seizure), appropriate treatment was provided and the trial was discontinued.

### 2.6 Functional MRI Analysis

Forty‐two participants completed the post‐treatment fMRI assessment; two were excluded during quality control, leaving a final imaging sample of 40 participants (Active‐cTBS, *n* = 21; Sham‐cTBS, *n* = 19). Structural and resting‐state functional MRI (rs‐fMRI) data were acquired on a 3‐T scanner. T1‐weighted structural images were obtained at 1 mm isotropic resolution, while rs‐fMRI used a single‐shot echo‐planar imaging sequence (TR/TE = 2000/30 ms, 3.5 × 3.5 × 4 mm^3^ voxels). Preprocessing and functional connectivity analysis were performed using the CONN toolbox (v22a) in MATLAB, including realignment, normalization to MNI space, segmentation, smoothing (8 mm FWHM), and denoising via aCompCor with bandpass filtering (0.008–0.09 Hz). ROI‐to‐ROI analysis involved 132 regions from the Harvard–Oxford and AAL atlases. Group comparisons (Active vs. Sham cTBS) were conducted using ANCOVA, controlling for age, gender, and symptom duration, with FDR correction for multiple comparisons. Quality control included motion assessment (framewise displacement threshold: 1.6 mm). Detailed methods are described in the File S2.

### 2.7 Statistical Analysis

Based on prior studies, the sample size was calculated using PASS version 15.0 (NCSS LLC, Kaysville, UT, USA). Assuming response rates (defined as a ≥ 3‐point reduction in PSQI score) of 70% in the active group and 30% in the sham group [35], 34 participants per group were required with 2‐sided *α* = 0.05, 90% power, and 20% attrition. Primary analyses were performed in the intention‐to‐treat (ITT) population and repeated in the per‐protocol (PP) population. In the primary ITT analysis, missing clinical data were imputed using last observation carried forward (LOCF), consistent with the original statistical analysis plan. Given the known limitations of LOCF, sensitivity analyses were additionally conducted using (1) linear mixed‐effects models fitted to the raw observed data without imputation and (2) multiple imputation by chained equations (MICE) with predictive mean matching for continuous clinical outcomes. Twenty imputed datasets were generated, analyzed separately, and pooled according to Rubin's rules. Consistency across these approaches was used to assess the robustness of the findings. Analyses of fMRI included only participants with complete imaging data, and safety analyses included all enrolled participants.

Continuous variables were summarized using descriptive statistics, including means and standard deviations (SDs), or medians and interquartile ranges (IQRs) or 95% confidence intervals (CIs), as appropriate. Categorical variables were presented as counts and percentages. Baseline characteristics were compared using the Student *t*‐test, Wilcoxon rank‐sum test, or Fisher's exact test, as appropriate. The primary endpoint was analyzed using logistic regression adjusted for age, sex, disease duration, and baseline PSQI score. Secondary clinical outcomes were analyzed using linear mixed‐effects models including post‐baseline visits (Weeks 2 and 6), with group, time, and group × time interaction as fixed effects, baseline value as a covariate, and participant as a random intercept. Adjusted between‐group differences were estimated from marginal means with 95% CIs; degrees of freedom were computed using the Satterthwaite method, and Holm correction was applied for multiple testing. Prespecified models included a baseline‐adjusted model and a fully adjusted model additionally controlling for age, sex, and disease duration. For the fMRI analyses, baseline, and post‐treatment between‐group ROI‐to‐ROI comparisons were performed using second‐level general linear models in CONN. The longitudinal group × time interaction was tested using the contrast (Active_post − Active_pre) − (Sham_post − Sham_pre). Age, sex, and symptom duration were included as covariates. Multiple comparisons were controlled using the false discovery rate procedure implemented in CONN, and FDR‐adjusted *p* < 0.05 was considered statistically significant. Analyses were performed in SPSS 23, R 4.3.0, and DPABI v8.3 in MATLAB. Detailed methods are described in the File S2.

## 3 Results

### 3.1 Baseline Characteristics of Patients With Insomnia Disorder

Between August 16, 2023 and September 29, 2024, a total of 92 patients with insomnia disorder were screened for eligibility, of whom 72 were randomized. Patients were allocated to either the Active‐cTBS group (*n* = 36) or the Sham‐cTBS group (*n* = 36). All randomized participants were included in the ITT analysis; three patients in the Sham‐cTBS group did not follow the protocol. Thus, 36 and 33 patients were included in the PP analysis (Figure 1). The ITT population had a mean (SD) age of 44.3 (11.6) years, and 51 participants (70.8%) were female. At baseline, the mean (SD) history of insomnia disorder was 5.4 (6.2) years, and the mean (SD) PSQI score was 13.5 (2.0) (Table 1, File S3, Table S2). Baseline demographic and clinical characteristics were balanced between the treatment groups in both the ITT (Table 1) and PP populations (File S3, Table S3).

**FIGURE 1:**
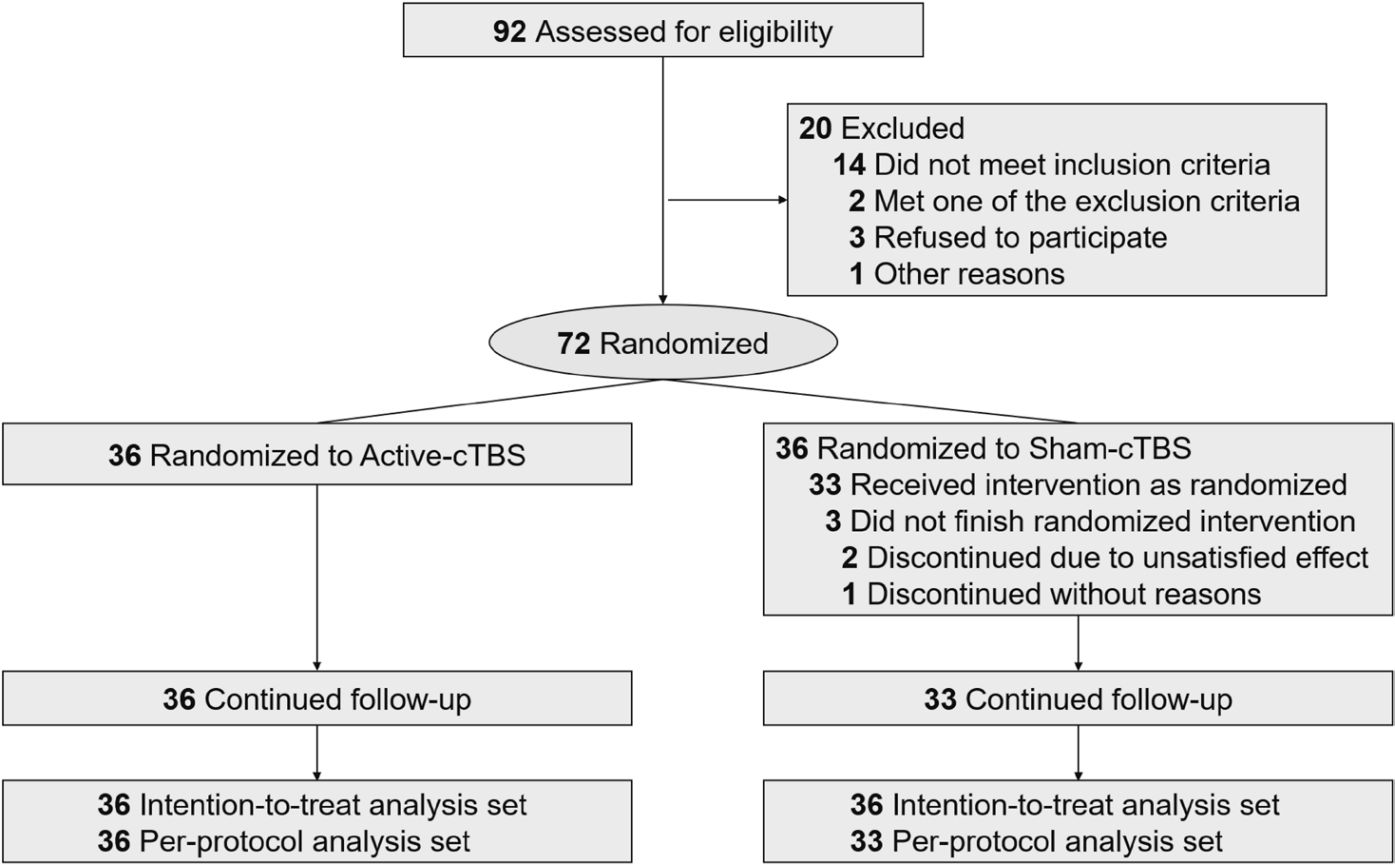
Flowchart of the trial.

**TABLE 1:** Baseline characteristics of the patients, intention‐to‐treat population.

	Total (*N* = 72)	Active‐cTBS (*N* = 36)	Sham‐cTBS (*N* = 36)	Statistic	*p*
Age, years, mean (SD)	44.3 (11.6)	41.7 (11.6)	45.8 (10.8)	−1.55	0.125
Gender (female), *n* (%)	51 (70.8%)	27 (75.0%)	24 (66.7%)	0.62	0.605
Disease duration, years, mean (SD)	5.4 (6.2)	5.6 (6.7)	5.2 (5.7)	0.27	0.786
BMI, mean (SD)	22.5 (2.7)	22.6 (3.0)	22.4 (2.3)	0.32	0.752
Complications, *n* (%)					
Hypertension	13 (18.1%)	7 (19.4%)	6 (16.7%)	—	1.000
Diabetes	3 (4.2%)	2 (5.6%)	1 (2.8%)	—	1.000
Insomnia severity					
PSQI, mean (SD)	13.5 (2.0)	13.2 (2.0)	13.8 (2.0)	−1.27	0.207
ISI, mean (SD)	18.7 (4.7)	19.1 (4.5)	18.4 (4.9)	0.63	0.530
ESS, mean (SD)	5.4 (4.9)	5.8 (5.1)	5.1 (4.8)	0.60	0.551
HAMD, mean (SD)	8.9 (3.3)	8.7 (3.5)	9.2 (3.1)	−0.65	0.523
HAMA, mean (SD)	8.8 (3.0)	8.5 (3.2)	9.2 (2.6)	−1.02	0.312

*Note:* Continuous variables were compared using the independent‐samples *t*‐test, and categorical variables were compared using the chi‐squared test or Fisher's exact test, as appropriate. Corresponding test statistics (*t* or *χ* ^2^) and *p* values are presented where applicable.

Abbreviations: BMI, body mass index; cTBS, continuous theta burst stimulation; ESS, Epworth Sleepiness Scale; HAMA, Hamilton Anxiety Scale; HAMD, Hamilton Depression Scale; ISI, Insomnia Severity Index; PSQI, Pittsburgh Sleep Quality Index; SD, standard deviation.

### 3.2 The Effects of Cerebellar cTBS on Insomnia Disorder Clinical Outcomes

Following the two‐week treatment period, a significantly greater proportion of patients in the Active‐cTBS group achieved a ≥ 3‐point improvement in PSQI scores compared with the Sham‐cTBS group (27/36 [75.0%] vs. 12/36 [33.3%], absolute difference: 41.7%, adjusted odds ratio: 6.06, 95% CI: 2.17–18.50; *p* < 0.001). This corresponds to a number needed to treat (NNT) of 2.4. Moreover, PSQI scores were significantly lower in the Active‐cTBS group than in the Sham‐cTBS group both at the post‐treatment assessment (adjusted mean difference = −3.34, 95% CI: −4.57 to −2.12; *p* < 0.001) and at the 1‐month follow‐up (adjusted mean difference = −3.70, 95% CI: −4.93 to −2.48; *p* < 0.001) (Figure 2, Table 2).

**FIGURE 2:**
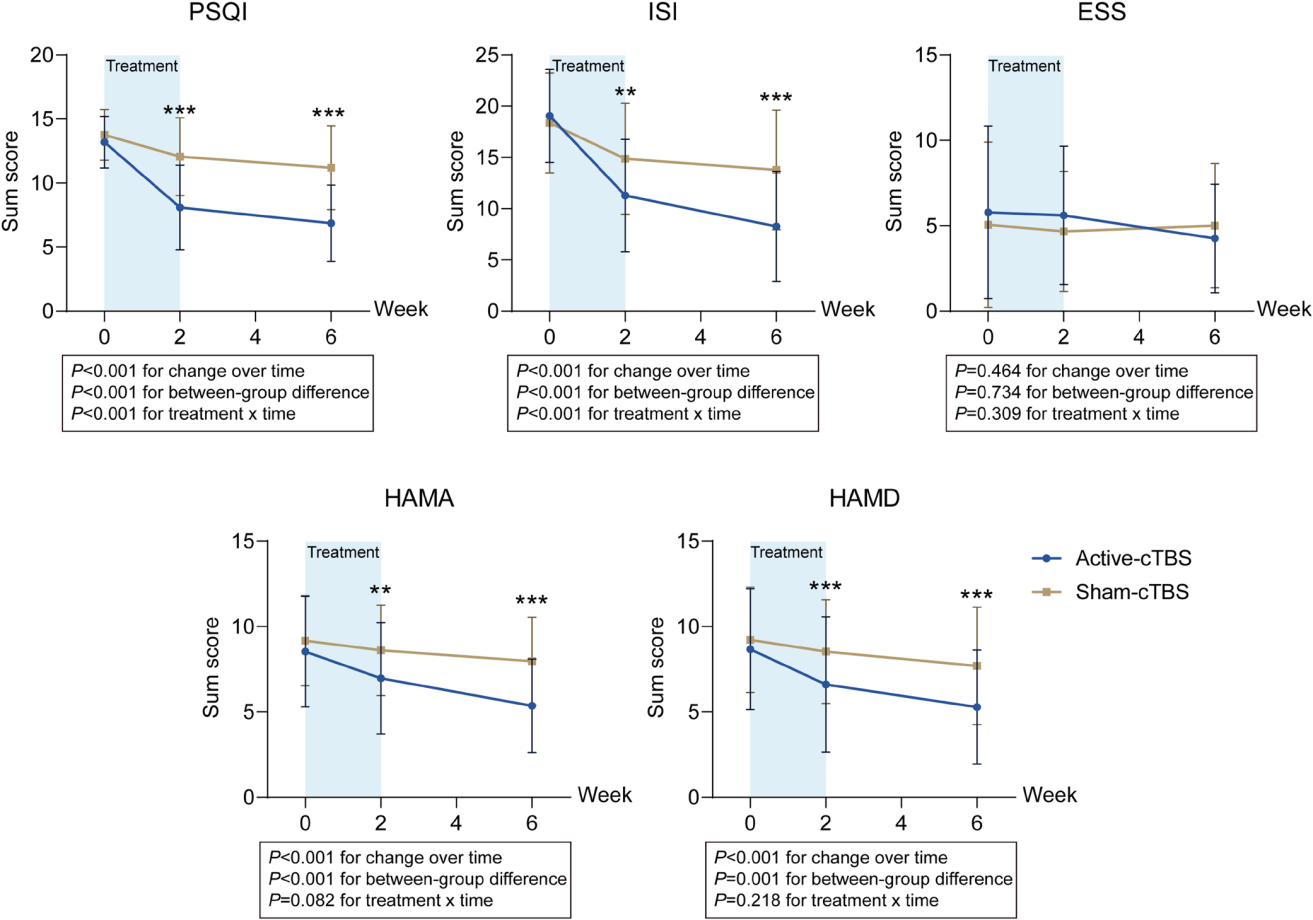
Clinical outcomes of patients. The scores of PSQI, ISI, ESS, HAMA, and HAMD at baseline, after 2‐week treatment, and after 1‐month follow‐up. ESS, Epworth Sleepiness Scale; HAMA, Hamilton Anxiety Scale; HAMD, Hamilton Depression Scale; ISI, Insomnia Severity Index; PSQI, Pittsburgh Sleep Quality Index. Statistically significant values: *p < 0.05, **p < 0.01, ***p < 0.001.

**TABLE 2:** Clinical outcomes of the intention‐to‐treat population.

	Active‐cTBS (*N* = 36)	Sham‐cTBS (*N* = 36)	Unadjusted analysis		Adjusted analysis	
			OR/Mean difference (95% CI)	*p*	OR/Mean difference (95% CI)	*p*
Primary outcome^a^						
PSQI score improved by ≥ 3 points	27/36 (75.0%)	12/36 (33.3%)	6.00 (2.22, 17.50)	< 0.001	6.06 (2.17, 18.50)	< 0.001
Secondary outcomes						
PSQI score^b^						
Baseline	13.19 (2.01)	13.75 (1.99)	—	—	—	—
2 week	8.08 (3.31)	12.06 (3.03)	−3.50 (−4.76, −2.24)	< 0.001	−3.34 (−4.57, −2.12)	< 0.001
6 week	6.86 (2.99)	11.19 (3.28)	−3.86 (−5.12, −2.60)	< 0.001	−3.70 (−4.93, −2.48)	< 0.001
ISI score^b^						
Baseline	19.06 (4.54)	18.36 (4.89)	—	—	—	—
2 week	11.28 (5.49)	14.86 (5.42)	−3.97 (−6.27, −1.67)	< 0.001	−3.60 (−5.89, −1.32)	0.002
6 week	8.25 (5.39)	13.78 (5.84)	−5.91 (−8.21, −3.61)	< 0.001	−5.55 (−7.83, −3.26)	< 0.001
ESS score^b^						
Baseline	5.78 (5.06)	5.06 (4.84)	—	—	—	—
2 week	5.61 (4.05)	4.67 (3.51)	0.57 (−0.62, 1.75)	0.346	0.48 (−0.74, 1.71)	0.436
6 week	4.25 (3.18)	5.00 (3.65)	−1.13 (−2.32, 0.06)	0.062	−1.21 (−2.44, 0.02)	0.053
HAMD score^b^						
Baseline	8.67 (3.55)	9.22 (3.09)	—	—	—	—
2 week	6.61 (3.97)	8.53 (3.04)	−1.40 (−2.15, −0.66)	< 0.001	−1.30 (−2.02, −0.57)	< 0.001
6 week	5.28 (3.34)	7.69 (3.44)	−1.90 (−2.65, −1.16)	< 0.001	−1.80 (−2.52, −1.07)	< 0.001
HAMA score^b^						
Baseline	8.53 (3.24)	9.17 (2.65)	—	—	—	—
2 week	6.97 (3.26)	8.61 (2.65)	−1.11 (−1.75, −0.46)	< 0.001	−0.98 (−1.62, −0.34)	0.003
6 week	5.36 (2.74)	7.97 (2.58)	−2.08 (−2.72, −1.44)	< 0.001	−1.95 (−2.59, −1.31)	< 0.001

*Note:* Data are presented as *n*/*N* (%) or mean (SD), as appropriate.

Abbreviations: CI, confidence interval; cTBS, continuous theta burst stimulation; ESS, Epworth Sleepiness Scale; HAMA, Hamilton Anxiety Scale; HAMD, Hamilton Depression Scale; ISI, Insomnia Severity Index; *N*, number; OR, odds ratio; PSQI, Pittsburgh Sleep Quality Index.

^a^ Logistic regression was used in the ITT population, with or without adjustment for sex, age, baseline score, and disease duration.

^b^ Linear mixed‐effects models were used in the ITT population, with or without adjustment for sex, age, and disease duration.

Similarly, patients in the Active‐cTBS group demonstrated significantly greater improvements in ISI, HAMA, and HAMD scores compared with the Sham‐cTBS group both after 2‐week treatment and at 1‐month follow‐up assessments (all *p* < 0.01). No significant differences in ESS scores were observed between groups at either the end of treatment (*p* = 0.436) or at the 1‐month follow‐up (*p* = 0.053; Figure 2, Table 2). Notably, significant treatment‐by‐time interaction effects were observed for both PSQI and ISI scores (both *p* < 0.001), indicating that the efficacy of Active‐cTBS increased over time compared with Sham‐cTBS (Figure 2). The results in the PP population were consistent with those observed in the ITT population (File S3, Table S4).

Sensitivity analyses for missing clinical data showed that the results were robust to different analytic approaches. The linear mixed‐effects models fitted to the raw observed data without LOCF imputation and the multiple‐imputation analyses using MICE with predictive mean matching yielded effect estimates and statistical inferences consistent with those of the primary analysis (File S3, Tables S5 and S6).

Adverse events were infrequent during active stimulation: 2 of 36 patients (5.6%) in the Active‐cTBS group reported mild headaches, mainly characterized by scalp tingling/discomfort during treatment sessions. These symptoms resolved spontaneously after each session, were tolerable, and did not lead to treatment interruption or withdrawal. No other adverse events were reported (File S3, Table S7).

### 3.3 fMRI Analysis

In the fMRI analysis, ROI‐to‐ROI functional connectivity (FC) was assessed in patients with complete imaging data (File S3, Table S8) to compare the Active‐cTBS and Sham‐cTBS groups at baseline and after the 2‐week intervention. No ROI‐to‐ROI FC differences between the Active‐cTBS and Sham‐cTBS groups survived FDR correction at baseline (File S4, Figure S1). However, at the post‐treatment assessment, significant group differences emerged. Specifically, compared with the Sham‐cTBS group, the Active‐cTBS group exhibited significantly decreased FC between the left postcentral gyrus (PostCG) and the right frontal operculum cortex (FO) (*t*(35) = −3.49, p‐FDR = 0.040), bilateral insular cortex (IC) (left: *t*(35) = −4.21, p‐FDR = 0.010; right: *t*(35) = −4.15, p‐FDR = 0.010), and bilateral central opercular cortex (CO) (left: *t*(35) = −3.44, p‐FDR = 0.040; right: *t*(35) = −4.10, p‐FDR = 0.010). Similarly, decreased FC was observed between the right PostCG and the right FO (*t*(35) = −3.58, p‐FDR = 0.033), bilateral IC (left: *t*(35) = −4.05, p‐FDR = 0.018; right: *t*(35) = −3.65, p‐FDR = 0.033), and the left CO (*t*(35) = −4.67, p‐FDR = 0.006). Furthermore, significantly decreased FC was also identified between the precuneus and the left frontal orbital cortex (FOrb) (*t*(35) = −3.95, p‐FDR = 0.047) (Figure 3A,B, File S3, Table S9). These findings represent between‐group differences observed at the post‐treatment assessment and should not be interpreted as evidence of differential pre‐to‐post changes.

**FIGURE 3:**
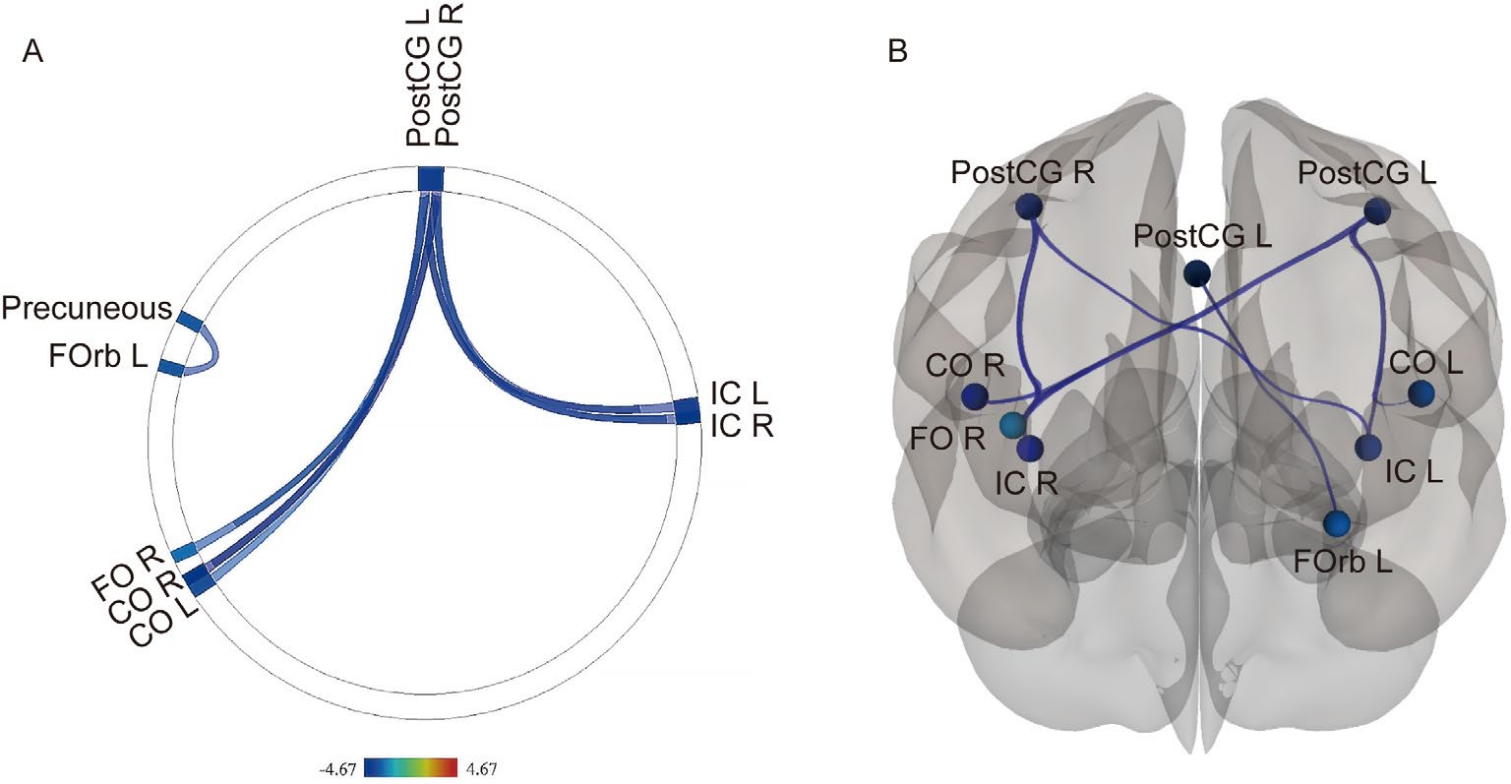
Post‐treatment between‐group differences in functional connectivity. (A) Diagram showing ROI‐to‐ROI functional connectivity differences between the Active‐cTBS and Sham‐cTBS groups at the post‐treatment assessment. (B) Three‐dimensional visualization of the same post‐treatment between‐group differences. CO, central opercular cortex; FC, functional connectivity; FO, frontal operculum cortex; FOrb, frontal orbital cortex; IC, insular cortex; L, left; PostCG, postcentral gyrus; R, right.

To directly assess differential longitudinal changes in functional connectivity, a longitudinal ROI‐to‐ROI analysis revealed a significant group × time interaction in FC between the right amygdala and right FOrb (*t*(35) = 4.02, uncorrected *p* < 0.001, p‐FDR = 0.038), with a greater pre‐to‐post increase in the Active‐cTBS group than in the Sham‐cTBS group. The direction of the interaction indicated a more positive pre‐to‐post change in the Active‐cTBS group than in the Sham‐cTBS group.

## 4 Discussion

Our study suggests that 2 weeks of neuronavigation‐guided cerebellar cTBS improved insomnia symptoms, as indicated by reduced PSQI and ISI scores, with benefits maintained at 1‐month follow‐up. The treatment was well tolerated, with no serious adverse events, supporting further investigation of the cerebellum as a potential neuromodulation target.

TMS has been widely studied for ID, targeting the posterior parietal cortex, temporal cortex, and most commonly the DLPFC [35, 36, 37]. Across studies, stimulation parameters, treatment duration, and targeted regions have varied considerably [37]. Therefore, comparisons between our findings and those of previous rTMS studies should be interpreted cautiously. The choice of 10 sessions was based on the literature and our group's prior TMS studies in epilepsy and disorders of consciousness [12, 13, 37]. In this study, active cerebellar cTBS reduced PSQI scores after 2 weeks, and the benefit persisted at 1‐month follow‐up, suggesting potential therapeutic value for insomnia. Although HAMA and HAMD scores differed between groups after treatment and follow‐up, the lack of a significant treatment × time interaction means its effects on anxiety and depressive symptoms remain unclear and should be interpreted cautiously. Moreover, the 1‐month follow‐up was short for a chronic condition like ID, and the long‐term effects of cerebellar cTBS remain unclear. A potential concern is that bilateral inhibitory cerebellar cTBS may cause mutual interference, similar to cancellation effects reported with bilateral cerebral stimulation, although this analogy should be interpreted cautiously. Interhemispheric competition in cortical stimulation is thought to depend largely on transcallosal interactions [38], whereas cerebellar stimulation primarily influences brain networks through cerebello‐thalamo‐cortical pathways. Moreover, insomnia is increasingly regarded as a disorder of hyperarousal and distributed network dysfunction rather than unilateral hemispheric imbalance, and neuroimaging studies have implicated abnormal cerebro‐cerebellar connectivity in this condition [14, 15, 16, 17, 23]. Importantly, according to our previous studies, we used alternating rather than simultaneous bilateral stimulation, which may further reduce the likelihood of direct cancellation. Our previous studies showed that alternating bilateral cerebellar TMS can be effective in drug‐resistant epilepsy [12] and disorders of consciousness [13], supporting the cerebellum as a potential therapeutic TMS target.

Our fMRI analyses provided exploratory evidence of post‐treatment between‐group differences. At the post‐treatment assessment, the Active‐cTBS group showed lower functional connectivity than the Sham‐cTBS group in connections involving the postcentral gyri, opercular‐insular regions, and the precuneus–frontal orbital cortex. These findings support the hyperarousal model of insomnia, suggesting that abnormal activity in the salience/interoceptive network and the default mode network (DMN) may contribute to heightened sensory, cognitive, and emotional arousal [39, 40, 41, 42]. Specifically, reduced FC between the postcentral gyrus and insular/opercular regions may indicate decreased arousal‐related network activity, while altered precuneus–FOrb connectivity may reflect reduced rumination and emotional arousal at sleep onset [43]. Given the roles of the precuneus in the DMN and the FOrb in emotional and arousal regulation, these connectivity changes may be associated with improved sleep quality [44, 45]. The observed functional connectivity changes, along with clinical improvement, suggest that connectivity remodeling may represent a candidate neural correlate of treatment response [41]. However, these connections did not show significant group × time interactions. They should therefore be interpreted specifically as between‐group differences observed at the post‐treatment assessment, rather than as evidence that active cTBS induced connectivity decreases or produced functional connectivity remodeling. These exploratory findings do not establish a causal mechanism of cTBS in insomnia. Because the imaging analyses were correlational, the observed FC changes should be considered candidate neural correlates of treatment response rather than causal evidence.

Importantly, the longitudinal analysis revealed a significant group × time interaction in FC between the right amygdala and the right frontal orbital cortex. This was the only connection showing an FDR‐corrected differential longitudinal effect. The direction of the interaction indicated a more positive pre‐to‐post change in the Active‐cTBS group than in the Sham‐cTBS group. The amygdala supports emotional and arousal processing [46], whereas the frontal orbital cortex is involved in top‐down emotional regulation [47, 48]. Thus, the greater increase in amygdala–prefrontal connectivity after active‐cTBS may indicate enhanced regulation of emotion‐ and arousal‐related circuits [48, 49]. As insomnia is associated with hyperarousal and impaired affective regulation [39], this finding may point to neural circuits potentially relevant to insomnia. However, because this exploratory analysis included only participants with complete fMRI data, the result should be interpreted cautiously and confirmed in larger samples.

In this study, we selected the cerebellum as a therapeutic target for insomnia disorder and applied cTBS to the CDN to modulate lobule VIIb [20, 50]. The cerebellum is increasingly recognized as an important hub in sleep regulation [17]. It expresses core clock genes involved in circadian rhythms, shows differential activity across sleep stages, and contributes to sleep–wake regulation [15, 16, 17, 51, 52, 53, 54, 55]. Anatomically, lobule VIIb projects through the CDN to the ventral lateral thalamus and then to distributed frontoparietal cortical regions [20, 56, 57, 58]. Because these cortical and subcortical structures are involved in sleep regulation, this pathway may support the use of cerebellar cTBS to influence sleep‐related brain networks in insomnia [17, 59, 60]. However, the fMRI findings are exploratory and correlational. Although post‐treatment between‐group differences and a group × time effect were observed, these findings do not establish causality or clarify the underlying neural mechanisms. Further studies are needed to elucidate the biological pathways linking the clinical and imaging findings.

Although the planned sample size was 68, 72 participants were ultimately enrolled because four additional individuals had already completed screening and provided informed consent when recruitment was nearing completion. They were retained to maintain study continuity. This decision was made before unblinding and data analysis. Sensitivity analyses using the first 68 participants and all 72 participants yielded consistent effect sizes, 95% confidence intervals, and *p* values, with no change in the statistical conclusions (File S3, Table S10). Baseline characteristics were also comparable between the two samples (all *p* > 0.05; File S3, Table S11), suggesting that inclusion of the additional participants did not introduce selection bias or compromise randomization.

This study has several limitations. As a single‐center trial with a short 1‐month follow‐up, its generalizability and long‐term effects require confirmation in larger multicenter studies. Although participants and outcome assessors were intended to be blinded, blinding integrity was not formally assessed. In addition, objective sleep measures were not included, so the results relied mainly on subjective outcomes. Future studies should evaluate the success of blinding and incorporate both subjective and objective sleep assessments. Post‐treatment fMRI data were unavailable for some patients because of refusal, head motion, or scheduling conflicts, although baseline characteristics remained comparable between groups (File S3, Table S12). We also did not examine correlations between FC changes and clinical improvement because the imaging sample may have been underpowered for these post hoc analyses. Moreover, the correlational nature of the clinical and fMRI findings precludes causal inference and warrants further imaging and mechanistic studies.

## 5 Conclusions

In conclusion, cerebellar cTBS was well tolerated and improved insomnia symptoms in the short term, with benefits still seen at 1‐month follow‐up. Exploratory neuroimaging revealed post‐treatment connectivity differences and a group × time effect, suggesting possible neural correlates rather than widespread connectivity changes. Larger multicenter randomized controlled trials with longer follow‐up and objective sleep measures are needed to confirm these findings.

## Author Contributions

Wen Jiang conceived and designed the study and provided the main administrative, technical, and material support. Feng Xia provided the administrative, technical, and material support, and contributed to the study design. Xin‐Yao Liu contributed to study design, enrolment of patients, data collection, and integrity of the data. Dong Dong, Chang‐Geng Song, and Yu‐Xuan Ma participated in study design, enrolment of patients, data collection and interpretation, and statistical analysis of data. Dian‐Wei Wu, Xuan Wang, Jian‐Min Hao, Hui Li, and Jing‐Jing Zhao provided support for data collection, statistical analysis, and interpretation of the data. Geng‐Yao Hu contributed to TMS treatment. Xin‐Yao Liu, Dong Dong, and Chang‐Geng Song drafted the manuscript with input from all the authors and with no external writing assistance. All authors have critically reviewed and approved the final version of the manuscript.

## Funding

The study was funded by the Clinical Research Program of the Fourth Military Medical University (XJZT2024LY21) and the National Natural Science Foundation of China (82471471; 82401702).

## Ethics Statement

This study was conducted in accordance with the principles of the Declaration of Helsinki. Written informed consent was obtained from all patients (or their parent/legal guardian/next of kin) prior to inclusion in the study. The study protocol was reviewed and approved by the Ethics Committee of Xijing Hospital, approval number KY20232190.

## Conflicts of Interest

The authors declare no conflicts of interest. We confirm that we have read the Journal's position on issues involved in ethical publication and affirm that this report is consistent with those guidelines.

## Supporting information


**File S1:** cns71231‐sup‐0001‐FileS1.docx.

**File S2:** cns71231‐sup‐0002‐SupplementaryFile2.docx.

**File S3:** cns71231‐sup‐0003‐SupplementaryFile3.docx.
**Table S1:** Inclusion and exclusion criteria.
**Table S2:** Demographic and clinical information of patients.
**Table S3:** Baseline demographics and clinical characteristics, per‐protocol population.
**Table S4:** Clinical outcomes of the per‐protocol population.
**Table S5:** Sensitivity analysis for missing clinical data to raw observed data without LOCF imputation.
**Table S6:** Sensitivity analysis for missing clinical data using multiple imputation by chained equations with predictive mean matching.
**Table S7:** Adverse events by group.
**Table S8:** Details of missing data in outcomes.
**Table S9:** Changes in functional connectivity between brain regions after the 2‐week treatment comparing active and sham stimulation groups.
**Table S10:** Sensitivity analysis of primary outcome for the calculated sample size of 68 cases versus the actual 72 subjects.
**Table S11:** Baseline data for the estimated sample size of 68 participants versus the actual 72 participants.
**Table S12:** Baseline characteristics of patients with vs. without post‐treatment fMRI data.

**File S4:** cns71231‐sup‐0004‐SupplementaryFile4.docx.
**Figure S1:** Uncorrected baseline between‐group ROI‐to‐ROI functional connectivity matrix (Active‐cTBS vs. Sham‐cTBS).

**Data S1:** CONSORT checklist.

## Data Availability

The study protocol, statistical analysis plan, and de‐identified participant data underlying this article's findings will be accessible for non‐commercial use, subject to executive committee approval of a research proposal, beginning 12 months after publication. For further details, please contact the corresponding authors by email.
