# Supplementary material for: Cerebellar Continuous Theta Burst Stimulation for Insomnia Disorder: A Randomized Sham‐Controlled Trial
Source: CNS Neurosci Ther. 2026 Oct 26;32(10):e71231. doi: 10.1002/cns.71231 (PMC13697236; doi:10.1002/cns.71231)
Supplement: Supplementary file 1 [file CNS-32-e71231-s001.docx]

**Supplementary Figure**

**Contents:**

**Supplementary Fig. S1.** Uncorrected baseline between-group ROI-to-ROI functional connectivity matrix (Active-cTBS vs. Sham-cTBS).

**Supplementary Fig. S1. Uncorrected baseline between-group ROI-to-ROI functional connectivity matrix (Active-cTBS vs. Sham-cTBS).** The 132 ROIs include: FP r (Frontal Pole Right); FP l (Frontal Pole Left); IC r (Insular Cortex Right); IC l (Insular Cortex Left); SFG r (Superior Frontal Gyrus Right); SFG l (Superior Frontal Gyrus Left); MidFG r (Middle Frontal Gyrus Right); MidFG l (Middle Frontal Gyrus Left); IFG tri r (Inferior Frontal Gyrus, pars triangularis Right); IFG tri l (Inferior Frontal Gyrus, pars triangularis Left); IFG oper r (Inferior Frontal Gyrus, pars opercularis Right); IFG oper l (Inferior Frontal Gyrus, pars opercularis Left); PreCG r (Precentral Gyrus Right); PreCG l (Precentral Gyrus Left); TP r (Temporal Pole Right); TP l (Temporal Pole Left); aSTG r (Superior Temporal Gyrus, anterior division Right); aSTG l (Superior Temporal Gyrus, anterior division Left); pSTG r (Superior Temporal Gyrus, posterior division Right); pSTG l (Superior Temporal Gyrus, posterior division Left); aMTG r (Middle Temporal Gyrus, anterior division Right); aMTG l (Middle Temporal Gyrus, anterior division Left); pMTG r (Middle Temporal Gyrus, posterior division Right); pMTG l (Middle Temporal Gyrus, posterior division Left); toMTG r (Middle Temporal Gyrus, temporooccipital part Right); toMTG l (Middle Temporal Gyrus, temporooccipital part Left); aITG r (Inferior Temporal Gyrus, anterior division Right); aITG l (Inferior Temporal Gyrus, anterior division Left); pITG r (Inferior Temporal Gyrus, posterior division Right); pITG l (Inferior Temporal Gyrus, posterior division Left); toITG r (Inferior Temporal Gyrus, temporooccipital part Right); toITG l (Inferior Temporal Gyrus, temporooccipital part Left); PostCG r (Postcentral Gyrus Right); PostCG l (Postcentral Gyrus Left); SPL r (Superior Parietal Lobule Right); SPL l (Superior Parietal Lobule Left); aSMG r (Supramarginal Gyrus, anterior division Right); aSMG l (Supramarginal Gyrus, anterior division Left); pSMG r (Supramarginal Gyrus, posterior division Right); pSMG l (Supramarginal Gyrus, posterior division Left); AG r (Angular Gyrus Right); AG l (Angular Gyrus Left); sLOC r (Lateral Occipital Cortex, superior division Right); sLOC l (Lateral Occipital Cortex, superior division Left); iLOC r (Lateral Occipital Cortex, inferior division Right); iLOC l (Lateral Occipital Cortex, inferior division Left); ICC r (Intracalcarine Cortex Right); ICC l (Intracalcarine Cortex Left); MedFC (Frontal Medial Cortex); SMA r (Juxtapositional Lobule Cortex -formerly Supplementary Motor Cortex- Right); SMA L(Juxtapositional Lobule Cortex -formerly Supplementary Motor Cortex- Left); SubCalC (Subcallosal Cortex); PaCiG r (Paracingulate Gyrus Right); PaCiG l (Paracingulate Gyrus Left); AC (Cingulate Gyrus, anterior division); PC (Cingulate Gyrus, posterior division); Precuneous (Precuneous Cortex); Cuneal r (Cuneal Cortex Right); Cuneal l (Cuneal Cortex Left); FOrb r (Frontal Orbital Cortex Right); FOrb l (Frontal Orbital Cortex Left); aPaHC r (Parahippocampal Gyrus, anterior division Right); aPaHC l (Parahippocampal Gyrus, anterior division Left); pPaHC r (Parahippocampal Gyrus, posterior division Right); pPaHC l (Parahippocampal Gyrus, posterior division Left); LG r (Lingual Gyrus Right); LG l (Lingual Gyrus Left); aTFusC r (Temporal Fusiform Cortex, anterior division Right); aTFusC l (Temporal Fusiform Cortex, anterior division Left); pTFusC r (Temporal Fusiform Cortex, posterior division Right); pTFusC l (Temporal Fusiform Cortex, posterior division Left); TOFusC r (Temporal Occipital Fusiform Cortex Right); TOFusC l (Temporal Occipital Fusiform Cortex Left); OFusG r (Occipital Fusiform Gyrus Right); OFusG l (Occipital Fusiform Gyrus Left); FO r (Frontal Operculum Cortex Right); FO l (Frontal Operculum Cortex Left); CO r (Central Opercular Cortex Right); CO l (Central Opercular Cortex Left); PO r (Parietal Operculum Cortex Right); PO l (Parietal Operculum Cortex Left); PP r (Planum Polare Right); PP l (Planum Polare Left); HG r (Heschl's Gyrus Right); HG l (Heschl's Gyrus Left); PT r (Planum Temporale Right); PT l (Planum Temporale Left); SCC r (Supracalcarine Cortex Right); SCC l (Supracalcarine Cortex Left); OP r (Occipital Pole Right); OP l (Occipital Pole Left); Thalamus r; Thalamus l; Caudate r; Caudate l; Putamen r; Putamen l; Pallidum r; Pallidum l; Hippocampus r; Hippocampus l; Amygdala r; Amygdala l; Accumbens r; Accumbens l; Brain-Stem; Cereb1 l (Cerebelum Crus1 Left); Cereb1 r (Cerebelum Crus1 Right); Cereb2 l (Cerebelum Crus2 Left); Cereb2 r (Cerebelum Crus2 Right); Cereb3 l (Cerebelum 3 Left); Cereb3 r (Cerebelum 3 Right); Cereb45 l (Cerebelum 4 5 Left); Cereb45 r (Cerebelum 4 5 Right); Cereb6 l (Cerebelum 6 Left); Cereb6 r (Cerebelum 6 Right); Cereb7 l (Cerebelum 7b Left); Cereb7 r (Cerebelum 7b Right); Cereb8 l (Cerebelum 8 Left); Cereb8 r (Cerebelum 8 Right); Cereb9 l (Cerebelum 9 Left); Cereb9 r (Cerebelum 9 Right); Cereb10 l (Cerebelum 10 Left); Cereb10 r (Cerebelum 10 Right); Ver12 (Vermis 1 2); Ver3 (Vermis 3); Ver45 (Vermis 4 5); Ver6 (Vermis 6); Ver7 (Vermis 7); Ver8 (Vermis 8); Ver9 (Vermis 9); Ver10 (Vermis 10).
