# Supplementary material for: Cerebellar Continuous Theta Burst Stimulation for Insomnia Disorder: A Randomized Sham‐Controlled Trial
Source: CNS Neurosci Ther. 2026 Oct 26;32(10):e71231. doi: 10.1002/cns.71231 (PMC13697236; doi:10.1002/cns.71231)
Supplement: Supplementary file 3 [file CNS-32-e71231-s003.docx]

**Supplementary Tables**

**Contents:**

**Table S1.** Inclusion and exclusion criteria.

**Table S2.** Demographic and clinical information of patients.

**Table S3.** Baseline demographics and clinical characteristics, per-protocol population.

**Table S4.** Clinical outcomes of the per-protocol population.

**Table S5.** Sensitivity analysis for missing clinical data to raw observed data without LOCF imputation.

**Table S6.** Sensitivity analysis for missing clinical data using multiple imputation by chained equations with predictive mean matching.

**Table S7.** Adverse events by group.

Table S8. Details of missing data in outcomes.

**Table S9.** Changes in functional connectivity between brain regions after the two-week treatment comparing active and sham stimulation groups.

**Table S10.** Sensitivity analysis of primary outcome for the calculated sample size of 68 cases versus the actual 72 subjects.

**Table S11.** Baseline data for the estimated sample size of 68 participants versus the actual 72 participants.

**Table S12.** Baseline characteristics of patients with vs. without post-treatment fMRI data.

**Table S1. Inclusion and exclusion criteria**

| Inclusion criteria | Exclusion criteria |
| --- | --- |
| 1. Age: 18-60 years old.  2. All participants met diagnostic criteria for insomnia disorder according to the Diagnostic and Statistical Manual of Mental Disorders-V (DSM-V), and scored ＞10 on PSQI, ≤14 on the Hamilton Anxiety Scale (HAMA), and ≤17 on the Hamilton Depression Scale (HAMD).  3. No sleep-affecting drugs were taken 1 month before or during the trial (sleep medication, benzodiazepines, hypnotics, or over-the-counter supplements and herbal sleep aids, etc.).  4. Patients can cooperate to complete the treatment and related examination items.  5. All subjects signed informed-consent forms before enrolling in the trial. | 1. The patient is unable to cooperate with the completion of treatment and examinations.  2. There are other types of sleep disorders.  3. A history of psychotropic drug or alcohol dependence or abuse in the last 2 years.  4. Have undertaken trans-meridian travel across more than two time zones or worked night shifts in the preceding month or during the trial period.  5. Planned surgical treatment within 1 month before or during the trial.  6. Evidence of neurological or other physical diseases such as respiratory, cardiac, renal, hepatic, endocrinal diseases, and other psychiatric diseases as assessed by clinical history, physical examination, or routine laboratory tests.  7. Pregnancy or breastfeeding women.  8. Any contraindication for TMS (including implanted metal and devices in the body, and history of epilepsy).  9. Concurrent other clinical trials.  10. Patient or family member withdraws informed consent. |

Abbreviations: DSM-V, Diagnostic and Statistical Manual of Mental Disorders-V; PSQI, Pittsburgh Sleep Quality Index; HAMD, Hamilton Depression Scale; HAMA, Hamilton Anxiety Scale; TMS, Transcranial magnetic stimulation

**Table S2. Demographic and clinical information of patients**

| Patient  No./Sex/Age | Group | Disease course, year | BMI | PSQI | RMT, μV |
| --- | --- | --- | --- | --- | --- |
| 1/M/37 | Active-cTBS | 7 | 22.40 | 11 | 35 |
| 2/F/41 | Active -cTBS | 5 | 21.30 | 14 | 28 |
| 3/F/60 | Sham -cTBS | 20 | 24.20 | 15 | 50 |
| 4/F/52 | Sham -cTBS | 3.5 | 22.10 | 12 | 45 |
| 5/F/54 | Sham -cTBS | 6 | 23.50 | 16 | 45 |
| 6/F/50 | Active -cTBS | 6 | 24.10 | 12 | 38 |
| 7/F/36 | Active -cTBS | 2 | 22.40 | 11 | 39 |
| 8/M/54 | Sham -cTBS | 20 | 22.06 | 16 | 40 |
| 9/M/57 | Active -cTBS | 0.83 | 26.67 | 15 | 38 |
| 10/F/46 | Active -cTBS | 10 | 25.39 | 17 | 40 |
| 11/M/36 | Sham -cTBS | 5 | 24.82 | 12 | 40 |
| 12/F/49 | Active -cTBS | 0.58 | 26.03 | 11 | 40 |
| 13/F/51 | Active -cTBS | 25 | 21.78 | 13 | 48 |
| 14/F/56 | Active -cTBS | 30 | 19.95 | 13 | 37 |
| 15/M/60 | Sham -cTBS | 1.5 | 22.23 | 11 | 36 |
| 16/F/51 | Active -cTBS | 20 | 21.64 | 13 | 39 |
| 17/F/43 | Sham -cTBS | 6 | 23.81 | 13 | 48 |
| 18/F/40 | Active -cTBS | 2 | 23.88 | 15 | 43 |
| 19/M/53 | Sham -cTBS | 0.83 | 29.06 | 15 | 42 |
| 20/M/37  21/F/38  22/F/49  23/F/20  24/M/27  25/F/36  26/F/60  27/M/41  28/F/60  29/F/51  30/M/59  31/F/58  32/M/57  33/M/40  34/F/52  35/M/53  36/F/31  37/F/56  38/F/33  39/M/58  40/F/36  41/F/56  42/F/51  43/M/32  44/M/24  45/F/44  46F/26  47/F/35  48/F/60  49/F/36  50/F/55  51/F/37  52/F/58  53/F/19  54/M/56  55/F/49  56/F/54  57/F/57  58/M/22  59/F/34  60/F/33  61/F/45  62/F/39  63/F/59  64/F/47  65/M/24  66/F/47  67/F/35  68/F/33  69/M/42  70/M/40  71/F/33  72/F/26 | Sham -cTBS  Active -cTBS  Sham -cTBS  Active -cTBS  Active -cTBS  Sham -cTBS  Sham -cTBS  Active -cTBS  Sham -cTBS  Sham -cTBS  Active -cTBS  Sham -cTBS  Active -cTBS  Active -cTBS  Sham -cTBS  Sham -cTBS  Active -cTBS  Active -cTBS  Sham -cTBS  Sham -cTBS  Sham -cTBS  Sham -cTBS  Sham -cTBS  Sham -cTBS  Active -cTBS  Sham -cTBS  Active -cTBS  Active -cTBS  Sham -cTBS  Active -cTBS  Sham -cTBS  Active -cTBS  Active -cTBS  Sham -cTBS  Sham -cTBS  Active -cTBS  Active -cTBS  Sham -cTBS  Active -cTBS  Sham -cTBS  Active -cTBS  Sham -cTBS  Sham -cTBS  Active -cTBS  Active -cTBS  Sham -cTBS  Active -cTBS  Sham -cTBS  Active -cTBS  Sham -cTBS  Active -cTBS  Sham -cTBS  Active -cTBS | 0.5  10  1.5  6  5  2  5  0.25  0.5  20  6  10  1  10  6  5  0.67  2  1  15  10  3  0.67  1  8  10  1  3  2  3  8  2  1  1  1  3  7  3  5  2  2  1  0.5  0.33  0.5  3  5  1  6  5  5  3  2 | 24.22  17.91  20.28  27.22  31.14  17.30  20.71  21.72  22.66  19.71  25.00  17.90  23.12  23.03  21.88  24.39  19.63  25.97  18.75  23.67  19.81  22.15  24.77  23.20  18.11  23.38  20.06  20.42  22.22  22.27  23.44  25.78  24.03  18.78  25.50  17.63  25.39  23.33  18.94  21.64  18.59  22.60  22.76  23.03  21.48  24.28  24.30  20.70  20.08  23.12  22.84  22.10  21.08 | 11  12  16  14  11  12  15  11  15  16  12  15  13  17  16  16  11  16  15  15  14  11  14  11  11  17  12  11  16  11  17  15  13  12  14  12  12  14  16  15  14  13  11  16  16  11  16  12  15  11  11  12  11 | 37  42  48  38  37  44  34  38  45  30  38  32  45  43  36  47  34  35  32  46  50  28  44  35  30  32  34  48  41  32  34  37  43  37  32  36  25  38  42  24  40  30  33  35  28  26  40  39  30  31  29  33  30 |

Abbreviations: cTBS, continuous theta burst stimulation; BMI, Body Mass Index; PSQI, Pittsburgh Sleep Quality Index; RMT, Resting Motor Threshold.

**Table S3. Baseline demographics and clinical characteristics, per-protocol population**

|  | **Total (*N*=69)** | **Active-cTBS (*N*=36)** | **Sham-cTBS (*N*=33)** | **Statistic** | ***P* value** |
| --- | --- | --- | --- | --- | --- |
| **Age, years, mean (SD)** | 43.8 (11.6) | 41.7 (11.6) | 46.0 (11.3) | -1.56 | 0.124 |
| **Gender (female), n (%)** | 48 (69.6%) | 27 (75.0%) | 21 (63.6%) | 1.05 | 0.304 |
| **Disease duration, years, mean (SD)** | 5.4 (6.3) | 5.6 (6.7) | 5.2 (5.9) | 0.27 | 0.790 |
| **BMI, mean (SD)** | 22.5 (2.7) | 22.6 (3.0) | 22.5 (2.4) | 0.15 | 0.878 |
| **Complications, n (%)** |  |  |  |  |  |
| Hypertension | 13 (18.8%) | 7 (19.4%) | 6 (18.2%) | 0.02 | 0.893 |
| Diabetes | 3 (4.3%) | 2 (5.6%) | 1 (3.0%) | - | 1.000 |
| **Insomnia severity** |  |  |  |  |  |
| PSQI, mean (SD) | 13.4 (2.0) | 13.2 (2.0) | 13.7 (2.1) | -1.01 | 0.317 |
| ISI, mean (SD) | 18.8 (4.8) | 19.1 (4.5) | 18.5 (5.1) | 0.52 | 0.606 |
| ESS, mean (SD) | 5.4 (5.0) | 5.8 (5.1) | 5.0 (5.0) | 0.66 | 0.513 |
| HAMD, mean (SD) | 8.9 (3.4) | 8.7 (3.6) | 9.2 (3.2) | -0.61 | 0.544 |
| HAMA, mean (SD) | 8.9 (3.0) | 8.5 (3.2) | 9.2 (2.7) | -0.98 | 0.332 |

Continuous variables were compared using the independent-samples t test, and categorical variables were compared using the chi-square test or Fisher’s exact test, as appropriate. Corresponding test statistics (t or χ²) and P values are presented where applicable. Abbreviations: cTBS, continuous theta burst stimulation; BMI, body mass index; PSQI, Pittsburgh Sleep Quality Index; ISI, Insomnia Severity Index; ESS, Epworth Sleepiness Scale; HAMD, Hamilton Depression Scale; HAMA, Hamilton Anxiety Scale; SD, standard deviation.

**Table S4. Clinical outcomes of the per-protocol population**

|  | Active-cTBS  (*N*=36) | Sham-cTBS  (*N*=33) | Unadjusted analysis | | Adjusted analysis | |
| --- | --- | --- | --- | --- | --- | --- |
|  |  |  | OR/Mean difference (95%CI) | *P* value | OR/Mean difference (95%CI) | *P* value |
| **Primary outcome** |  |  |  |  |  |  |
| PSQI score improved by ≥ 3 points^a^ | 27 /36(75.0%) | 12/33 (36.4%) | 5.25 (1.86, 14.79) | 0.002 | 5.23 (1.79, 15.32) | 0.003 |
| **Secondary outcomes** |  |  |  |  |  |  |
| **PSQI score**^b^ |  |  |  |  |  |  |
| Baseline | 13.19 (2.01) | 13.73 (2.07) | - | - | - | - |
| 2 week | 8.08 (3.31) | 11.88 (3.10) | -3.35 (-4.64, -2.05) | ＜0.001 | -3.16 (-4.41, -1.90) | ＜0.001 |
| 6 week | 6.86 (2.99) | 10.94 (3.30) | -3.63 (-4.92, -2.33) | ＜0.001 | -3.44 (-4.70, -2.18) | ＜0.001 |
| **ISI score**^b^ |  |  |  |  |  |  |
| Baseline | 19.06 (4.54) | 18.48 (5.05) | - | - | - | - |
| 2 week | 11.28 (5.49) | 14.67 (5.58) | -3.71 (-6.08, -1.34) | 0.003 | -3.33 (-5.70, -0.97) | 0.006 |
| 6 week | 8.25 (5.39) | 13.48 (5.98) | -5.55 (-7.93, -3.18) | ＜0.001 | -5.18 (-7.54, -2.81) | ＜0.001 |
| **ESS score**^b^ |  |  |  |  |  |  |
| Baseline | 5.78 (5.06) | 5.00 (4.97) | - | - | - | - |
| 2 week | 5.61 (4.05) | 4.58 (3.54) | 0.63 (-0.60, 1.87) | 0.309 | 0.55 (-0.73, 1.83) | 0.397 |
| 6 week | 4.25 (3.18) | 4.94 (3.69) | -1.09 (-2.32, 0.14) | 0.082 | -1.18 (-2.46, 0.10) | 0.071 |
| **HAMD score**^b^ |  |  |  |  |  |  |
| Baseline | 8.67 (3.55) | 9.24 (3.20) | - | - | - | - |
| 2 week | 6.61 (3.97) | 8.48 (3.14) | -1.34 (-2.11, -0.57) | ＜0.001 | -1.22 (-1.97, -0.47) | 0.002 |
| 6 week | 5.28 (3.34) | 7.58 (3.55) | -1.77 (-2.54, -0.99) | ＜0.001 | -1.65 (-2.40, -0.90) | ＜0.001 |
| **HAMA score**^b^ |  |  |  |  |  |  |
| Baseline | 8.53 (3.24) | 9.21 (2.73) | - | - | - | - |
| 2 week | 6.97 (3.26) | 8.61 (2.75) | -1.06 (-1.73, -0.40) | 0.002 | -0.91 (-1.58, -0.25) | 0.007 |
| 6 week | 5.36 (2.74) | 7.91 (2.66) | -1.98 (-2.64, -1.31) | ＜0.001 | -1.83 (-2.49, -1.16) | ＜0.001 |

Data are presented as n/N (%) or Mean (SD), as appropriate.

^a^ Logistic regression was used in the PP population, with or without adjustment for sex, age, baseline score, and disease duration.

^b^ Linear mixed-effects models were used in the PP population, with or without adjustment for sex, age, and disease duration.

Abbreviations: cTBS, continuous theta burst stimulation; PSQI, Pittsburgh Sleep Quality Index; ISI, Insomnia Severity Index; ESS, Epworth Sleepiness Scale; HAMD, Hamilton Depression Scale; HAMA, Hamilton Anxiety Scale; N, number; OR, Odds Ratio; CI, confidence interval.

**Table S5. Sensitivity analysis for missing clinical data to raw observed data without LOCF imputation.**

|  | Active-cTBS  (*N*=36) | Sham-cTBS  (*N*=33) | Unadjusted analysis | | Adjusted analysis | |
| --- | --- | --- | --- | --- | --- | --- |
|  |  |  | OR/Mean difference (95%CI) | *P* value | OR/Mean difference (95%CI) | *P* value |
| **Primary outcome** |  |  |  |  |  |  |
| PSQI score improved by ≥ 3 points^a^ | 27 /36(75.0%) | 12/33 (36.4%) | 5.25 (1.86, 14.79) | 0.002 | 5.23 (1.79, 15.32) | 0.003 |
| **Secondary outcomes** |  |  |  |  |  |  |
| **PSQI score**^b^ |  |  |  |  |  |  |
| Baseline | 13.19 (2.01) | 13.73 (2.07) | - | - | - | - |
| 2 week | 8.08 (3.31) | 11.88 (3.10) | -3.35 (-4.64, -2.05) | ＜0.001 | -3.16 (-4.41, -1.90) | ＜0.001 |
| 6 week | 6.86 (2.99) | 10.94 (3.30) | -3.63 (-4.92, -2.33) | ＜0.001 | -3.44 (-4.70, -2.18) | ＜0.001 |
| **ISI score**^b^ |  |  |  |  |  |  |
| Baseline | 19.06 (4.54) | 18.48 (5.05) | - | - | - | - |
| 2 week | 11.28 (5.49) | 14.67 (5.58) | -3.71 (-6.08, -1.34) | 0.003 | -3.33 (-5.70, -0.97) | 0.006 |
| 6 week | 8.25 (5.39) | 13.48 (5.98) | -5.55 (-7.93, -3.18) | ＜0.001 | -5.18 (-7.54, -2.81) | ＜0.001 |
| **ESS score**^b^ |  |  |  |  |  |  |
| Baseline | 5.78 (5.06) | 5.00 (4.97) | - | - | - | - |
| 2 week | 5.61 (4.05) | 4.58 (3.54) | 0.63 (-0.60, 1.87) | 0.309 | 0.55 (-0.73, 1.83) | 0.397 |
| 6 week | 4.25 (3.18) | 4.94 (3.69) | -1.09 (-2.32, 0.14) | 0.082 | -1.18 (-2.46, 0.10) | 0.071 |
| **HAMD score**^b^ |  |  |  |  |  |  |
| Baseline | 8.67 (3.55) | 9.24 (3.20) | - | - | - | - |
| 2 week | 6.61 (3.97) | 8.48 (3.14) | -1.34 (-2.11, -0.57) | ＜0.001 | -1.22 (-1.97, -0.47) | 0.002 |
| 6 week | 5.28 (3.34) | 7.58 (3.55) | -1.77 (-2.54, -0.99) | ＜0.001 | -1.65 (-2.40, -0.90) | ＜0.001 |
| **HAMA score**^b^ |  |  |  |  |  |  |
| Baseline | 8.53 (3.24) | 9.21 (2.73) | - | - | - | - |
| 2 week | 6.97 (3.26) | 8.61 (2.75) | -1.06 (-1.73, -0.40) | 0.002 | -0.91 (-1.58, -0.25) | 0.007 |
| 6 week | 5.36 (2.74) | 7.91 (2.66) | -1.98 (-2.64, -1.31) | ＜0.001 | -1.83 (-2.49, -1.16) | ＜0.001 |

Data are presented as n/N (%) or Mean (SD), as appropriate.

^a^ Logistic regression was used in the PP population, with or without adjustment for sex, age, baseline score, and disease duration.

^b^ Linear mixed-effects models were used in the PP population, with or without adjustment for sex, age, and disease duration.

Abbreviations: cTBS, continuous theta burst stimulation; PSQI, Pittsburgh Sleep Quality Index; ISI, Insomnia Severity Index; ESS, Epworth Sleepiness Scale; HAMD, Hamilton Depression Scale; HAMA, Hamilton Anxiety Scale; N, number; OR, Odds Ratio; CI, confidence interval.

**Table S6. Sensitivity analysis for missing clinical data using multiple imputation by chained equations with predictive mean matching.**

|  | Active-cTBS  (*N*=36) | Sham-cTBS  (*N*=36) | Unadjusted analysis | | Adjusted analysis | |
| --- | --- | --- | --- | --- | --- | --- |
|  |  |  | OR/Mean difference (95%CI) | *P* value | OR/Mean difference (95%CI) | *P* value |
| **Primary outcome** |  |  |  |  |  |  |
| PSQI score improved by ≥ 3 points^a^ | 27 /36(75.0%) | 13/36 (36.1%) | 5.31 (1.98, 15.30) | 0.001 | 5.19 (1.88, 15.50) | 0.002 |
| **Secondary outcomes** |  |  |  |  |  |  |
| **PSQI score**^b^ |  |  |  |  |  |  |
| Baseline | 13.19 (2.01) | 13.75 (1.99) | - | - | - | - |
| 2 week | 8.08 (3.31) | 11.92 (3.10) | -3.83 (-5.31, -2.36) | ＜0.001 | -3.45 (-4.78, -2.11) | ＜0.001 |
| 6 week | 6.86 (2.99) | 10.92 (3.16) | -4.06 (-5.53, -2.58) | ＜0.001 | -3.67 (-5.00, -2.33) | ＜0.001 |
| **ISI score**^b^ |  |  |  |  |  |  |
| Baseline | 19.06 (4.54) | 18.36 (4.89) | - | - | - | - |
| 2 week | 11.28 (5.49) | 14.89 (5.39) | -3.61 (-6.22, -1.00) | 0.007 | -3.20 (-5.66, -0.74) | 0.011 |
| 6 week | 8.25 (5.39) | 13.36 (6.03) | -5.11 (-7.72, -2.50) | ＜0.001 | -4.70 (-7.16, -2.24) | ＜0.001 |
| **ESS score**^b^ |  |  |  |  |  |  |
| Baseline | 5.78 (5.06) | 5.06 (4.84) | - | - | - | - |
| 2 week | 5.61 (4.05) | 4.53 (3.46) | 1.08 (-0.60, 2.76) | 0.203 | 0.97 (-0.73, 2.68) | 0.258 |
| 6 week | 4.25 (3.18) | 4.83 (3.57) | -0.58 (-2.26, 1.10) | 0.491 | -0.69 (-2.39, 1.01) | 0.421 |
| **HAMD score**^b^ |  |  |  |  |  |  |
| Baseline | 8.67 (3.55) | 9.22 (3.09) | - | - | - | - |
| 2 week | 6.61 (3.97) | 8.50 (3.01) | -1.89 (-3.51, -0.27) | 0.023 | -1.72 (-3.34, -0.10) | 0.037 |
| 6 week | 5.28 (3.34) | 7.64 (3.43) | -2.36 (-3.98, -0.74) | 0.005 | -2.19 (-3.81, -0.58) | 0.009 |
| **HAMA score**^b^ |  |  |  |  |  |  |
| Baseline | 8.53 (3.24) | 9.17 (2.65) | - | - | - | - |
| 2 week | 6.97 (3.26) | 8.53 (2.69) | -1.56 (-2.91, -0.20) | 0.025 | -1.34 (-2.66, -0.02) | 0.047 |
| 6 week | 5.36 (2.74) | 7.72 (2.83) | -2.36 (-3.72, -1.00) | ＜0.001 | -2.14 (-3.46, -0.83) | 0.002 |

Data are presented as n/N (%) or Mean (SD), as appropriate.

^a^ Logistic regression was used in the PP population, with or without adjustment for sex, age, baseline score, and disease duration.

^b^ Linear mixed-effects models were used in the PP population, with or without adjustment for sex, age, and disease duration.

Abbreviations: cTBS, continuous theta burst stimulation; PSQI, Pittsburgh Sleep Quality Index; ISI, Insomnia Severity Index; ESS, Epworth Sleepiness Scale; HAMD, Hamilton Depression Scale; HAMA, Hamilton Anxiety Scale; N, number; OR, Odds Ratio; CI, confidence interval.

**Table S7.** **Adverse events by group**

| **AEs/SAEs** | **Active-cTBS**  **(*N*=36)** | **Sham-cTBS**  **(*N*=36)** |
| --- | --- | --- |
| **Mild headache/ /scalp discomfort** | 2 | 0 |
| **severe headache** | 0 | 0 |
| **Dizziness** | 0 | 0 |
| **Tinnitus** | 0 | 0 |
| **seizure** | 0 | 0 |
| **Any treatment interruption due to adverse events** | 0 | 0 |
| **Withdrawal due to adverse events** | 0 | 0 |

Abbreviations: cTBS, continuous theta burst stimulation; N, number; AEs, adverse events; SAEs, serious adverse events.

Table S8. **Details of missing data in outcomes.**

|  | W0 | W2 | W6 |
| --- | --- | --- | --- |
| PSQI |  |  |  |
| Active-cTBS, patients with missing values, n | 0 | 0 | 0 |
| Sham-cTBS, patients with missing values, n | 0 | 3 | 3 |
| Total number of missing values, n | 0 | 3 | 3 |
| ESS |  |  |  |
| Active -cTBS, patients with missing values, n | 0 | 0 | 0 |
| Sham-cTBS, patients with missing values, n | 0 | 3 | 3 |
| Total number of missing values, n | 0 | 3 | 3 |
| ISI |  |  |  |
| Active -cTBS, patients with missing values, n | 0 | 0 | 0 |
| Sham-cTBS, patients with missing values, n | 0 | 3 | 3 |
| Total number of missing values, n | 0 | 3 | 3 |
| HAMA |  |  |  |
| Active -cTBS, patients with missing values, n | 0 | 0 | 0 |
| Sham-cTBS, patients with missing values, n | 0 | 3 | 3 |
| Total number of missing values, n | 0 | 3 | 3 |
| HAMD |  |  |  |
| Active -cTBS, patients with missing values, n | 0 | 0 | 0 |
| Sham-cTBS, patients with missing values, n | 0 | 3 | 3 |
| Total number of missing values, n | 0 | 3 | 3 |
| MRI |  |  |  |
| Active-cTBS, patients with missing values, n | 0 | 15 | - |
| Sham-cTBS, patients with missing values, n | 1 | 15 | - |
| Total number of missing values, n | 1 | 30 | - |

Numbers in each cell denote the exact number of missing values of that outcome on that visit. Abbreviations: PSQI, Pittsburgh Sleep Quality Index; ISI, Insomnia Severity Index; ESS, Epworth Sleepiness Scale; HAMD, Hamilton Depression Scale; HAMA, Hamilton Anxiety Scale; MRI, Magnetic resonance imaging; cTBS, continuous theta burst stimulation.

**Table S9. Changes in functional connectivity between brain regions after the two-week treatment comparing active and sham stimulation groups.**

| ROI 1 | ROI 2 | t value | *p*-FDR | *p-*uncorrected |
| --- | --- | --- | --- | --- |
| PostCG L | IC L | t (35) = -4.21 | 0.010 | < 0.001 |
|  | IC R | t (35) = -4.15 | 0.010 | < 0.001 |
|  | CO R | t (35) = -4.10 | 0.010 | < 0.001 |
|  | FO R | t (35) = -3.49 | 0.040 | 0.001 |
|  | CO L | t (35) = -3.44 | 0.040 | 0.002 |
| PostCG R | CO R | t (35) = -4.67 | 0.006 | < 0.001 |
|  | IC L | t (35) = -4.05 | 0.018 | < 0.001 |
|  | IC R | t (35) = -3.65 | 0.033 | < 0.001 |
|  | FO R | t (35) = -3.58 | 0.033 | < 0.001 |
| Precuneous | FOrb L | t (35) = -3.95 | 0.047 | < 0.001 |

The outcomes were analyzed by covariance, with age, gender, and symptom duration included as covariates. The analysis was performed across 132 whole-brain ROIs, corrected for multiple comparisons using the false discovery rate (FDR) across all ROI-to-ROI connections. Abbreviations: L, left; R, right; PostCG, postcentral gyrus; IC, insular cortex; CO, central opercular cortex; FO, frontal operculum cortex; Precuneous, precuneous cortex; FOrb, frontal orbital cortex.

|  | Active-cTBS | Sham-cTBS | Unadjusted analysis | | Adjusted analysis | |
| --- | --- | --- | --- | --- | --- | --- |
|  |  |  | OR (95% CI) | *P* value | OR (95% CI) | *P* value |
| **Primary outcome^a^** |  |  |  |  |  |  |
| PSQI score improved by ≥ 3 points, **n = 72** | 27/36 (75.0%) | 12/36 (33.3%) | 6.00 (2.15, 16.71) | 0.001 | 6.11 (2.12, 17.63) | 0.001 |
| PSQI score improved by ≥ 3 points, **n = 68** | 25/34 (73.5%) | 12/34 (35.3%) | 4.49 (1.58, 12.75) | 0.005 | 4.91 (1.61, 14.96) | 0.005 |

**Table S10.** **Sensitivity analysis of primary outcome for the calculated sample size of 68 cases versus the actual 72 subjects.**

Data are presented as n/N (%) or Mean (SD), as appropriate.

**^a^** Logistic regression was used in the ITT population, with or without adjustment for sex, age, and baseline score.

Abbreviations: cTBS, continuous theta burst stimulation; PSQI, Pittsburgh Sleep Quality Index; OR, Odds Ratio; CI, confidence interval.

**Table S11.** **Baseline data for the estimated sample size of 68 participants versus the actual 72 participants**

|  | ***N*=72** | ***N*=68** | ***P value*** |
| --- | --- | --- | --- |
| **Age, years, mean (SD)** | 44.3 (11.6) | 44.8 (11.6) | 0.787 |
| **Gender (female), n (%)** | 51 (70.8%) | 49 (72.1%) | 0.866 |
| **Disease duration, years, mean (SD)** | 5.4 (6.2) | 5.5 (6.3) | 0.928 |
| **BMI, mean (SD)** | 22.5 (2.7) | 22.5 (2.7) | 0.976 |
| **Complications, n (%)** |  |  |  |
| Hypertension | 13 (18.1%) | 13 (19.1%) | 0.873 |
| Diabetes | 3 (4.2%) | 3 (4.4%) | 1.000 |
| **Insomnia severity** |  |  |  |
| PSQI, mean (SD) | 13.5 (2.0) | 13.6 (2.0) | 0.702 |
| ISI, mean (SD) | 18.7 (4.7) | 18.7 (4.8) | 0.973 |
| ESS, mean (SD) | 5.4 (4.9) | 5.4 (5.0) | 0.995 |
| HAMD, mean (SD) | 8.9 (3.3) | 9.0 (3.3) | 0.880 |
| HAMA, mean (SD) | 8.8 (3.0) | 8.9 (3.0) | 0.921 |

Abbreviations: cTBS, continuous theta burst stimulation; BMI, Body Mass Index; PSQI, Pittsburgh Sleep Quality Index; ISI, Insomnia Severity Index; ESS, Epworth Sleepiness Scale; HAMD, Hamilton Depression Scale; HAMA, Hamilton Anxiety Scale; SD, Standard Deviation.

**Table S12. Baseline characteristics of patients with vs. without post-treatment fMRI data.**

|  | **With post-treatment fMRI**  ***N*=42** | **Without post-treatment fMRI**  ***N*=30** | ***P value*** |
| --- | --- | --- | --- |
| **Age, years, mean (SD)** | 42.3 (12.5) | 44.8 (11.6) | 0.387 |
| **Gender (female), n (%)** | 32 (76.2%) | 19 (63.3%) | 0.240 |
| **Disease duration, years, mean (SD)** | 5.5 (6.4) | 4.5 (4.9) | 0.446 |
| **BMI, mean (SD)** | 22.2 (2.4) | 23.0 (3.0) | 0.232 |
| **Insomnia severity** |  |  |  |
| PSQI, mean (SD) | 13.5 (2.1) | 13.3 (2.0) | 0.686 |
| ISI, mean (SD) | 18.9 (4.5) | 18.9 (5.5) | 1.000 |
| ESS, mean (SD) | 5.5 (5.4) | 5.1 (4.1) | 0.724 |
| HAMD, mean (SD) | 8.5 (3.2) | 9.9 (3.7) | 0.099 |
| HAMA, mean (SD) | 8.5 (2.7) | 9.5 (3.3) | 0.171 |

Abbreviations: cTBS, continuous theta burst stimulation; BMI, Body Mass Index; PSQI, Pittsburgh Sleep Quality Index; ISI, Insomnia Severity Index; ESS, Epworth Sleepiness Scale; HAMD, Hamilton Depression Scale; HAMA, Hamilton Anxiety Scale; SD, Standard Deviation.
