# Supplementary material for: Cerebellar Continuous Theta Burst Stimulation for Insomnia Disorder: A Randomized Sham‐Controlled Trial
Source: CNS Neurosci Ther. 2026 Oct 26;32(10):e71231. doi: 10.1002/cns.71231 (PMC13697236; doi:10.1002/cns.71231)
Supplement: Supplementary file 4 [file CNS-32-e71231-s004.docx]

**Supplementary Methods**

**Methods**

**Randomization and masking**

Participants were randomly assigned in a 1:1 ratio to either the active cerebellar continuous theta burst stimulation (Active-cTBS) group or the sham stimulation (Sham-cTBS) group. Randomization was conducted using a computer-generated schedule (SAS Statistical Package, version 9.2). Allocation sequences were generated and maintained by an independent, unmasked statistician who was not involved in any other aspect of the study. Group assignments were concealed from participants and all investigators involved in recruitment, assessment, and data analysis. The TMS operator, who was solely responsible for delivering the intervention within the assigned group, had access to group allocation but was not involved in outcome evaluation or data analysis. Outcome assessment was performed by one trained assessor who remained blinded to group allocation/intervention status throughout the study. The assessor followed a standardized evaluation procedure. Because only one assessor was involved, inter-rater reliability was not applicable. Blinding integrity was not formally assessed, and participants were not asked to guess treatment allocation after the intervention.

**Functional MRI Analysis**

***MRI data acquisition***

Forty-two participants completed the post-treatment fMRI assessment. Two participants were excluded during fMRI quality control, leaving 40 participants in the final imaging sample (Active-cTBS, n = 21; Sham-cTBS, n = 19). Structural MRI and resting-state fMRI (rs-fMRI) were conducted using a 3-T MR scanner (uMR 780, United Imaging Healthcare, Shanghai, China). T1-weighted images were acquired using the following parameters: echo time (TE), 3.1 ms; repetition time (TR), 7.2 ms; inversion time (TI), 750 ms; flip angle, 10°; slice thickness, 1 mm; 192 slices; matrix size, 256 × 256 mm^2^; voxel size, 1 × 1 × 1 mm³. Prior to the rs-fMRI scan, participants were asked to keep their eyes closed throughout the duration of the scan. rs-fMRI data were acquired using echo-planar images (single-shot) with the following parameters: TR/TE, 2000/30 ms; FA, 90°; FOV, 224× 224mm; matrix size, 64 × 64 mm^2^; slice thickness, 4 mm; 35 slices; voxel size, 3.5 × 3.5 × 4 mm³; and scan time, 8 min.

***Software Configuration & Functional Connectivity Analysis***

To ensure comparability and analytical rigor, all MRI analyses were conducted under consistent conditions and using standardized methods. We employed the same software version and adhered to a unified analysis framework for data interpretation and summarization. Specifically, all data were processed using the CONN toolbox (version 22a; Gabrieli Lab, MIT McGovern Institute for Brain Research, Cambridge, MA, USA; https://web.conn-toolbox.org) implemented in MATLAB R2024a (MathWorks, Natick, MA, USA). To minimize inter-individual variability and fluctuations in functional connectivity (FC), denoising procedures were applied using CONN's default preprocessing pipeline, which includes anatomical component-based noise correction (aCompCor). Confounding factors regressed out included signals from white matter, cerebrospinal fluid (CSF), realignment parameters, and scrubbing (outlier detection). Motion-related artifacts and physiological noise were also mitigated during this step. Subsequently, rs-fMRI data were temporally bandpass filtered at 0.008–0.09 Hz to reduce the influence of low-frequency scanner drift and high-frequency physiological noise such as respiratory and cardiac fluctuations.

***Preprocessing of MRI data***

Preprocessing of MRI data was conducted using the CONN toolbox. Functional images were first realigned to the anatomical reference and unwrapped to correct for geometric distortions. Slice-timing correction was applied, and outlier volumes were identified using scrubbing procedures based on the Artifact Detection Toolbox (ART). Both functional and structural images were normalized to the standard Montreal Neurological Institute (MNI) space and segmented into gray matter, white matter, and cerebrospinal fluid compartments. Functional data were then spatially smoothed with an 8-mm full-width at half-maximum (FWHM) Gaussian kernel. To ensure data quality and reliability, a series of quality control procedures were implemented. These included visual inspection for artifacts and anatomical abnormalities, as well as motion assessment using framewise displacement (FD). Scans with FD values exceeding 1.6 mm were excluded from further analysis.

***Selection of regions of interest (ROIs) & ROI-to-ROI analyses***

Given that sleep regulation involves a wide range of brain regions across cortical and subcortical structures, we aimed to adopt a comprehensive approach in selecting ROIs. To ensure sufficient anatomical coverage and standardization, we utilized the default atlas provided by the CONN functional connectivity toolbox, which is derived from the Harvard-Oxford cortical and subcortical structural atlases and the AAL cerebellar atlas (132 ROIs). This standardized parcellation enabled consistent ROI definition across participants and ensured the inclusion of brain areas potentially involved in sleep-related processes. For the ROI-to-ROI statistical analyses, an analysis of covariance (ANCOVA) was conducted to compare FC between the active-cTBS and sham-cTBS groups at baseline and post-treatment stages, controlling for age, gender, and symptom duration as covariates. The analyses included 132 whole-brain ROIs, corrected for multiple comparisons using the false discovery rate (FDR) method across all ROI-to-ROI connections.

**Statistical Analysis**

Based on prior studies, the sample size was calculated using PASS version 15.0 (NCSS, LLC, Kaysville, UT, USA). Assuming response rates (defined as a ≥3-point reduction in PSQI score) of 70% in the active group and 30% in the sham group ^1^, 34 participants per group were required with 2-sided α=0.05, 90% power, and 20% attrition.

Primary analyses were performed in the intention-to-treat (ITT) population and repeated in the per-protocol (PP) population. In the primary ITT analysis, missing clinical data were imputed using last observation carried forward (LOCF), consistent with the original statistical analysis plan. Given the known limitations of LOCF, sensitivity analyses were additionally conducted using (1) linear mixed-effects models fitted to the raw observed data without imputation and (2) multiple imputation by chained equations (MICE) with predictive mean matching for continuous clinical outcomes. Twenty imputed datasets were generated, analyzed separately, and pooled according to Rubin’s rules. Consistency across these approaches was used to assess the robustness of the findings. Analyses of fMRI data were limited to participants who completed the examinations. Safety assessments were conducted in all enrolled patients.

Continuous variables were summarized using descriptive statistics, including means and standard deviations (SDs), or medians and interquartile ranges (IQRs) or 95% confidence intervals (CIs), as appropriate. Categorical variables were presented as counts and percentages. Comparisons of baseline clinical characteristics between groups were performed using the Student t-test, Wilcoxon rank-sum test, or Fisher’s exact test, as appropriate. For the primary efficacy endpoint, logistic regression analysis was employed to compare the proportion of patients achieving a ≥ 3-point improvement in PSQI score after treatment, adjusted for age, gender, disease duration, and baseline score. Secondary clinical outcomes were analyzed using a linear mixed model with baseline as a covariate. The model included only post-baseline measurements at 2 weeks (immediately post-treatment) and 6 weeks (one month post-treatment) as the dependent variable, with treatment group, time point, and their two-way interaction as fixed effects. Baseline measurement of the outcome was included as a primary covariate to adjust for chance imbalances between groups at randomization, and patient identifier was included as a random intercept to account for within-patient correlation between repeated measurements. Adjusted mean differences (95% confidence intervals) between the active treatment and sham groups at each post-baseline time point were estimated using estimated marginal means (EMMs). The Satterthwaite method was used to estimate degrees of freedom, and the Holm method was applied to adjust p-values for multiple comparisons. Two sets of analyses were prespecified: an unadjusted model that controlled only for baseline outcome measurement, and a fully adjusted model that further controlled for age, gender, and disease duration in addition to baseline outcome. For fMRI ROI analyses, Subject-level ROI-to-ROI functional connectivity estimates were analyzed using second-level general linear models in CONN. Baseline and post-treatment data were first compared between groups at each assessment. Differential longitudinal changes were then examined using the group × time contrast (Active_post − Active_pre) − (Sham_post − Sham_pre), with age, sex, and symptom duration included as covariates. Multiple comparisons were controlled using the false discovery rate procedure implemented in CONN, with FDR-adjusted P < 0.05 considered statistically significant. Analyses were performed using SPSS (version 23), R (version 4.3.0), and the DPABI toolbox (v8.3) in MATLAB.

**References**

1.	Huang Z, Li Y, Bianchi MT, et al. Repetitive transcranial magnetic stimulation of the right parietal cortex for comorbid generalized anxiety disorder and insomnia: A randomized, double-blind, sham-controlled pilot study. *Brain Stimul.* 2018;11(5):1103-1109.
