# Supplementary material for: Cerebellar Continuous Theta Burst Stimulation for Insomnia Disorder: A Randomized Sham‐Controlled Trial
Source: CNS Neurosci Ther. 2026 Oct 26;32(10):e71231. doi: 10.1002/cns.71231 (PMC13697236; doi:10.1002/cns.71231)
Supplement: Supplementary file 5 [file CNS-32-e71231-s005.docx]

**Protocol**

**Cerebellar continuous theta burst stimulation for insomnia disorder: a randomized sham-controlled trial**

**This supplementary file contains the following items:**

Part 1: Clinical Trial Protocol

Part 2: Statistical Analysis Plan

Part 3: Informed Consent Form

**Clinical Trial Protocol (Version 1.0)**

| **Full title of the trial** | Cerebellar continuous theta burst stimulation for insomnia disorder: a randomized sham-controlled trial |
| --- | --- |
| **Version and date of protocol** | Version 1.0, 15/05/2023 |
| **Trial device** | Neuro-MS/D Advanced Therapeutic Stimulator (Neurosoft) Neuronavigation System, Brain Science Tools BV (Utrecht) |
| **Trial site** | Xijing Hospital |
| **Primary investigator** | Prof. Wen Jiang, Department of Neurology, Xijing Hospital, Fourth Military Medical University, Xi'an 710032, Shaanxi, China |

**Abbreviations**

| **abbreviation** | **Full name** |
| --- | --- |
| ID | insomnia disorder |
| TBS | Theta-burst stimulation |
| cTBS | continuous theta burst stimulation |
| CRB | cerebellum |
| DSM-V | Diagnostic and Statistical Manual of Mental Disorders-V |
| CDN | cerebellum dentate nucleus |
| CBT-I | cognitive behavioral therapy for insomnia |
| TMS | transcranial magnetic stimulation |
| rTMS | repetitive transcranial magnetic stimulation |
| CRB-cTBS | continuous theta burst stimulation targeting cerebellar |
| DLPFC | Dorsal Lateral Prefrontal Cortex |
| MEP | motor-evoked potentials |
| iTBS | intermittent theta burst stimulation |
| DMN | default mode network |
| SN | salience network |
| REM | rapid eye movement sleep |
| non-REM | non-rapid eye movement sleep |
| PSQI | Pittsburgh Sleep Quality Index |
| ESS | Epworth Sleepiness Scale |
| ISI | insomnia Severity Index |
| HAMD | Hamilton Depression Scale |
| HAMA | Hamilton Anxiety Scale |
| MRI | Magnetic resonance imaging |
| GCP | Good Clinical Practice |
| RMT | resting motor threshold |
| BOLD | blood oxygen-level dependent |
| PI | Principal Investigator |
| IQR | interquartile range |
| SD | standard deviation |
| PP | Per-Protocol |
| EC | Executive Committee |
| QCAC | Quality Control and Assurance Committee |

**1 Summary**

| **English study title** | Cerebellar continuous theta burst stimulation for insomnia disorder: a randomized sham-controlled trial |
| --- | --- |
| **Trial Registration** | Registration Authority: ClinicalTrials.gov  Registration Number: NCT05992285  Registration Time: 15 August 2023 |
| **Trial device** | Neuro-MS/D Advanced Therapeutic stimulator (Neurosoft) Neuronavigation System, Brain Science Tools BV (Utrecht) |
| **Study design** | Randomized, controlled clinical trial |
| **Primary objectives** | To explore the efficacy of continuous theta burst stimulation (cTBS) on the cerebellar dentate nucleus in treating insomnia disorder (ID). |
| **Planned trial sites** | Single site |
| **Sample size** | 68 participants were randomized in a 1:1 ratio to Active-cTBS group or Sham-cTBS group |
| **Study population** | Adults with ID |
| **Main inclusion / Exclusion criteria:** | **Inclusion:**   1. Age: 18-60 years old. 2. All participants met diagnostic criteria for insomnia disorder according to the Diagnostic and Statistical Manual of Mental Disorders-V (DSM-V) and scored ＞10 on PSQI, ≤14 on the Hamilton Anxiety Scale (HAMA), and ≤17 on the Hamilton Depression Scale (HAMD). 3. No sleep-affecting drugs were taken 1 month before or during the trial (non-prescription sleep medication, benzodiazepines or hypnotics, etc.) 4. Patients can cooperate to complete the treatment and related examination items. 5. All subjects signed informed-consent forms before enrolling in the trial.   **Exclusion:**   1. The patient is unable to cooperate with the completion of treatment and examinations. 2. There are other types of sleep disorders. 3. A history of psychotropic drug or alcohol dependence or abuse in the last 2 years. 4. Have undertaken trans-meridian travel across more than two time zones or worked night shifts in the preceding month or during the trial period. 5. Plan surgical treatment within 1 month before or during the trial. 6. Evidence of neurological or other physical diseases such as respiratory, cardiac, renal, hepatic, endocrinal, and other psychiatric diseases as assessed by clinical history, physical examination, or routine laboratory tests. 7. Pregnancy or breastfeeding women. 8. Any contraindication for TMS (including implanted metal and devices in the body and history of epilepsy). 9. Concurrent other clinical trials. 10. Patient or family member withdraws informed consent. |
| **Study Treatments** | Active-cTBS group: 5 times per week for 2 weeks of Active-cTBS.  Sham-cTBS group: 5 times per week for 2 weeks of Sham-cTBS. |
| **Primary outcome** | The percentage of PSQI score improvement ≥3 points compared with baseline after the end of 10 treatments. |
| **Statistical analysis** | - Patients will be analyzed according to the principle of intention-to-treat (ITT) with the SPSS (version 23), R (version 4.3.0) and the DPABI toolbox (v8.3) in MATLAB. - We will compare results between the Active-cTBS and Sham-cTBS groups using appropriate statistical tests. For the main outcome, we will use logistic regression adjusting for gender, age, baseline score, and disease duration. Secondary outcomes will be analyzed with a mixed model, adjusting for baseline and considering patient differences. - We will conduct a covariance analysis on fMRI data. - Two-sided *P* values < 0.05 will be considered significant. |

**2 Introduction**

Insomnia disorder (ID) is a significant public health problem worldwide, with nearly a third of the general population experiencing insomnia symptoms in their lifetime [1]. ID refers to a subjective experience of inadequate sleep duration and/or quality that interferes with daily social functioning despite suitable sleep opportunities and environments. The main symptoms are difficulty falling asleep (latency more significant than 30 minutes), sleep maintenance disorders (≥2 awakenings throughout the night), early awakenings, decreased sleep quality, reduced total sleep time (usually < 6.5 hours), and daytime dysfunction. Daytime dysfunction due to insomnia often includes fatigue, low mood, physical discomfort, and cognitive impairment. Insomnia includes short-term insomnia (< 3 months) and chronic insomnia (≥3 months) according to the course of the disease. Studies have shown that sleep deprivation caused by ID not only seriously affects the quality of life but also leads to many health problems, such as cardiovascular disease, obesity, diabetes, immune system dysfunction, and cognitive and emotional disorders [2].

At present, the treatment for ID mainly includes drug therapy and non-drug therapy. The above treatments all have certain limitations. Although clinical trials have confirmed the short-term efficacy of drug therapy for insomnia, the long-term application still needs to bear the potential risks of adverse drug reactions (daydreaming, drowsiness, etc.), addiction, symptoms after drug withdrawal, and so on [3]. Non-drug treatment includes psychotherapy, physical therapy, and traditional Chinese medicine therapy. Psychotherapy mainly includes sleep hygiene education and cognitive behavioral therapy for insomnia (CBT-I). Although CBT is an effective method to treat ID, it has a long treatment duration, the cost is high, and the patient is not easy to adhere to. Based on the above, it is urgent to find a safe, effective, and easy non-drug treatment for insomnia.

Repetitive transcranial magnetic stimulation (rTMS) generates an induced current using a time-varying magnetic field and acts as a pulsed magnetic field on the cerebral cortex, depolarizing cortical neurons and producing action potentials, thereby affecting the electrical activity of the cerebral cortex [3]. Originally, Barker et al. applied rTMS to assess motor cortex-evoked potentials [4]. Nowadays, rTMS has been used in various degrees in many neurological and psychiatric diseases and has broad application prospects for the treatment of insomnia disorder [5]. The left Dorsal Lateral Prefrontal Cortex (DLPFC) is a key target area for TMS in treating insomnia. The earliest studies on the treatment of insomnia with rTMS found that it significantly reduced the recurrence rate and the recurrence rate within 3 months in the rTMS treatment group compared with the drug treatment group and the psychotherapy group [6]. Recent studies suggest that rTMS can improve sleep quality, optimize sleep structure, and maintain therapeutic effects better than drug therapy and CBT [7].

To date, the mechanism by which to treat insomnia disorder using rTMS stimulation is unclear. The mainstream view is that the cerebral cortex of ID is in a state of high arousal [8]. Therefore, low-frequency rTMS (≤1hz) can directly hyperpolarize nerve cells through pulsed magnetic fields and inhibit the overexcited cerebral cortex. Modular rTMS is an improved stimulation mode. Theta-burst stimulation (TBS) is commonly used for theta stimulation, characterized by bundle stimulation. Each bundle usually contains three pulses; the intra-bundle pulse frequency is 50Hz, and the interbundle pulse frequency is 5Hz. Continuous stimulation (cTBS) mainly evoked inhibitory effects on the cortex, and the inhibition of motor-evoked potentials (MEP) could last for 60 minutes, which was more durable than traditional rTMS-evoked potentials. Still, the intensity and time of stimulation were lower and shorter [9]. Intermittent theta burst stimulation (iTBS) can increase the excitability of the motor cortex. Recent studies have found that the iTBS program has been proven to be an effective, safe, tolerable, and fast-acting therapy for the treatment of refractory depression [10]. Insomnia disorder is a risk factor for depression, and previous studies have suggested that depression and insomnia disorder may share a common genetic background and biological mechanism [11]. As for the clinical research of cTBS therapy for ID, there are currently no relevant reports.

The abnormal connectivity of default mode network (DMN) and salience network (SN) in ID patients is related to subjective insomnia disorder, excessive arousal, emotional maladjustment, and emotional state integration disorder [12]. The limbic circuit is thought to play a crucial role in enhancing the recall of negative experiences, and previous studies have found that patients with ID have significantly reduced gray matter in the right mid-frontal gyrus [13]. These results suggest that a wide range of functional network abnormalities may play a role in the onset of ID. Until now, insomnia research has focused on the correlation between the neocortex and subcortical structures, while cerebellar activity has been largely ignored. However, studies have shown that cerebellar dysfunction not only impinges on motor control and motor memory formation but may also lead to alterations in sleep-wake cycles and even cause insomnia disorder [14]. The cerebellum (CRB) is well connected to the brain and the neuronal circuits involved in sleep-wake regulation [15]. In addition, many cerebellar neurons express clock genes associated with circadian rhythm control. However, research on the relationship between cerebellar function and different sleep stages is lacking. In earlier animal studies, it was found that cats' cerebellar crawling fibers and mossy fibers showed relatively low and high levels of activity during non-rapid eye movement sleep (non-REM) and rapid eye movement sleep (REM) periods, respectively, suggesting the existence of sleep-stage-dependent cerebellar activity [16, 17]. The cerebellum has strong interconnections with the cerebral cortex via the thalamus, pontine, mesencephalic diencephalic junction, and lower olive, and network activity in this cycle is heavily influenced by sleep-wake states [18,19]. Previous studies have shown that after implantation of electrodes in the cerebellar nucleus of epilepsy patients, the parietal nucleus and dentate nucleus show synchronous sharp discharge during the non-REM period and slight sharp potential during the REM period, suggesting that the cerebellar nucleus may be involved in the regulation of insomnia disorder [20]. Stimulation of Purkinje cells enhanced the cerebellum's inhibitory fibrous projection to the thalamic neural network and subsequently weakened its excitatory projection to the cerebral cortex. The mechanism of high-frequency rTMS targeting the cerebellum for the treatment of ID is unclear. The only efferent pathway of the cerebellum is inhibitory fiber projection from Purkinje cells to the deep cerebellum nucleus mass, and then widespread projection to the frontal and subcortical structures. The inhibitory output of Purkinje cells and extensive cortical projection support the possible role of cerebellar stimulation in reducing excitatory activity in the cerebral cortex. rTMS can excite Purkinje cells, and enhance cerebellar inhibitory fiber projection to thalamic neural network through long-term enhancement effects, and subsequently weaken excitatory projection to the cerebral cortex, reduce cortical excitability, and improve insomnia.

In conclusion, rTMS has non-trauma characteristics, good safety, repeatability, etc. Stimulating the cerebellar lobule VII, oriented toward the cerebellar dentate nucleus (CDN), can effectively reduce cortical excitability and improve insomnia. Therefore, this study intends to apply the cTBS mode to target cerebellar lobule VII, oriented toward the CDN for the first time in the treatment of ID and use the navigation system combined with magnetic resonance imaging to guide the positioning, hoping to further explore the efficacy and safety of rTMS and related technologies by improving and optimizing them.

**3 Objectives**

**3.1** **Primary objective**

The primary efficacy outcome was the proportion of participants with a ≥ 3-point improvement in PSQI scores from baseline (W0) after 10 treatments (W2）.

**3.2 Secondary objectives**

We will observe the changes in scores on the Pittsburgh Sleep Quality Index (PSQI), Epworth Sleepiness Scale (ESS), insomnia Severity Index (ISI), Hamilton Depression Scale (HAMD), and Hamilton Anxiety Scale (HAMA) after 10 sessions of cTBS treatment and at 6-week follow-up. The magnetic resonance imaging (MRI) changes was investigated.

**4** **Trial design**

**4.1** **Overall design**

This study will be a randomized, parallel-controlled clinical study—patients with ID who met the inclusion and exclusion criteria provided written informed consent. Patients will be randomly divided into the Active-cTBS and Sham-cTBS groups in a 1:1 ratio. They were treated 5 times a week for 2 weeks, 10 times in total, and followed up for 4 weeks after treatment.

**4.2** **Schematic diagram(s) of overall trial design**

**4.3 Determination of the sample size**

Based on the primary outcome of proportional improvement (reduction of 3 points or more) in the PSQI score in previous studies, the proportion of improvements was approximately 70% after TMS treatment and approximately 30% in the control group [21]. The estimated sample size was 34 participants per group, with a type I error (α) of 5%, a power of 1 − β of 0.1, and a dropout rate of 20%.

**5** **Selection of subjects**

**5.1** **Inclusion criteria**

1.	Age: 18-60 years old

2.	All participants met diagnostic criteria for insomnia disorder according to the Diagnostic and Statistical Manual of Mental Disorders-V (DSM-V) and scored ＞10 on PSQI, ≤14 on the Hamilton Anxiety Scale (HAMA), and ≤17 on the Hamilton Depression Scale (HAMD)

3.	No sleep-affecting drugs were taken 1 month before or during the trial (non-prescription sleep medication, benzodiazepines or hypnotics, etc.)

4.	Patients can cooperate to complete the treatment and related examination items

5.	All subjects signed informed-consent forms before enrolling in the trial.

**5.2** **Exclusion criteria:**

1.	The patient is unable to cooperate with the completion of treatment and examinations

2.	There are other types of sleep disorders

3.	A history of psychotropic drug or alcohol dependence or abuse in the last 2 years

4.	Have undertaken trans-meridian travel across more than two time zones or worked night shifts in the preceding month or during the trial period

5.	Plan surgical treatment within 1 month before or during the trial

6.	Evidence of neurological or other physical diseases such as respiratory, cardiac, renal, hepatic, endocrinal diseases, and other psychiatric diseases as assessed by clinical history, physical examination, or routine laboratory tests

7.	Pregnancy or breastfeeding women

8.	Any contraindication for TMS (including implanted metal and devices in the body and history of epilepsy)

9.	Concurrent other clinical trials

10.	Patient or family member withdraws informed consent.

**5.3** **Concomitant medication**

This study will not use ID-related drugs for treatment, and patients will be allowed to use basic disease medications, such as antihypertensive and hypoglycemic drugs.

**6** **Recruitment**

We will recruit patients from the neurology clinic at Xijing Hospital, Xi'an, Shaanxi Province, China. Patients attending routine follow-up appointments will be informed of the trial by their neurologists. Each candidate will be pre-screened according to the inclusion and exclusion criteria.

**7** **Study procedures and schedule of assessments**

**7.1** **Informed consent procedure**

All candidates will be assessed for their capacity to provide informed consent by an experienced neuropsychologist independent of the trial group. After a full explanation of the study's purpose, methodology, expected benefits, and potential risks, and if the patient (or legal guardian) is deemed capable of providing informed consent, the principal investigator or a person designated by the investigator will obtain written informed consent from each subject before participating in the trial.

All candidates will be informed of the trial's purpose and the possible risks and benefits. All researchers will receive up-to-date Good clinical practice (GCP) training. The investigator or his designee will explain that the patient is not obligated to participate in the trial and can withdraw without reason at any time during the trial.

We will provide the participants with a copy of the signed Informed Consent Form and retain the original signed form at the study site. Use only informed consent forms approved by the relevant clinical trial ethics committee. Each patient who wishes to participate in the trial and provides informed consent will be evaluated to determine if inclusion and exclusion criteria are met. Each patient's medical record must be carefully reviewed to review treatment history and ensure that the patient meets all inclusion criteria and does not meet any exclusion criteria. This review will take place after the patient has signed informed consent.

The informed consent form will be reviewed and updated as needed if new safety information results in a material change in the risk/benefit assessment. All subjects, including those who have been treated, will be informed of the new information provided with a revised informed consent form and asked to agree to continue participating in the study.

**7.2** **Screening and Qualification Assessment**

Contact information for the research team will be provided to each patient as part of the informed consent form. Basic information about each patient will be recorded, including sex, age, height, weight, family history, time of onset, Pittsburgh Sleep Quality Index Scale (PSQI), Epworth Sleepiness Scale (ESS), Insomnia Severity Index (ISI), Hamilton Depression Scale (HAMD), Hamilton Anxiety Scale (HAMA), and MRI. If patients meet the inclusion criteria, they will receive cTBS stimulation (or sham stimulation) for 2 weeks (10 sessions) and complete a 6-week follow-up.

**7.3** **Baseline assessments**

At the baseline, insomnia severity testing, psychological testing, and MRI will be used as baseline assessments for ID patients who meet the inclusion criteria (PSQI, ESS, ISI, HAMD, HAMA, MRI measures).

**7.4** **Parameters of Active-cTBS and Sham-cTBS**

**Cortical resting motor threshold (RMT):** The patient will be in a sitting position. Use the single pulse mode to stimulate the M1 cortex area of the thumb motor area on the hand side 10 times, of which 5 times can induce the thumb abductor muscle movement. The stimulation intensity was RMT.

**cTBS treatment parameters:** 50Hz, stimulation intensity of 80% RMT, duration of 40s as a group of stimulation, 600 stimulation pulses, repeated stimulation of bilateral cerebellar dentate nuclei in two groups, with a 5-minute interval between each group, 1200 stimulation pulses per site, 5 times a week, treatment for 2 weeks, then follow up at week 6. Stimulation was performed using a Neuro-MS/D stimulator (Neurosoft, GTMEDI Technology Co., Ltd., Beijing, China) connected to a double-cone coil mounted tangentially on the occipital scalp (external coil loop diameter, 125 mm; peak magnetic field, 4 Tesla).

**Sham treatment parameters:** Sham-cTBS was applied using the same parameters but with the coil held so that the edge was in contact with the head perpendicular to the scalp while the remainder was rotated 90°away from the scalp in the sagittal plane to reproduce the noise of the stimulation.

**Navigation:** A frameless neural navigator system (Brain Science Tools BV, Utrecht, The Netherlands; www.neuralnavigator.com) will be used to ensure consistent coil targeting of the cerebellar. It will reconstruct three-dimensional images based on individual pre-stimulation anatomical brain MRI scans. Continued feedback will be provided throughout the entire stimulation session via the navigation system to ensure precise targeting.

**7.5** **Randomization procedures**

The subjects will be randomly divided into Active-cTBS and Sham-cTBS groups on a 1:1 ratio. The randomization process was conducted by an independent investigator, who assigned each subject a treatment random number strictly in chronological order of enrollment.

**7.6 Blinding**

The TMS operator, who was solely responsible for delivering the intervention within the assigned group, had access to group allocation but was not involved in outcome evaluation or data analysis. Outcome assessment was performed by one trained assessor who remained blinded to group allocation/intervention status throughout the study. The assessor followed a standardized evaluation procedure. Because only one assessor was involved, inter-rater reliability was not applicable.

**7.7 MRI analysis**

**Functional MRI Analysis**

Images of structural MRI (T1-weighted imaging) will be performed at W0. The functional MRI (fMRI) (blood oxygen level dependent functional magnetic resonance imaging [BOLD]) was performed at W0 and W2.

***MRI data acquisition***

Structural MRI and resting-state fMRI (rs-fMRI) were conducted using a 3-T MR scanner (uMR 780, United Imaging Healthcare, Shanghai, China). T1-weighted images were acquired using the following parameters: echo time (TE), 3.1 ms; repetition time (TR), 7.2 ms; inversion time (TI), 750 ms; flip angle, 10°; slice thickness, 1 mm; 192 slices; matrix size, 256 × 256 mm^2^; voxel size, 1 × 1 × 1 mm³. Prior to the rs-fMRI scan, participants were asked to keep their eyes closed throughout the duration of the scan. rs-fMRI data were acquired using echo-planar images (single-shot) with the following parameters: TR/TE, 2000/30 ms; FA, 90°; FOV, 224× 224mm; matrix size, 64 × 64 mm^2^; slice thickness, 4 mm; 35 slices; voxel size, 3.5 × 3.5 × 4 mm³; and scan time, 8 min.

***Software Configuration & Functional Connectivity Analysis***

To ensure comparability and analytical rigor, all MRI analyses were conducted under consistent conditions and using standardized methods. We employed the same software version and adhered to a unified analysis framework for data interpretation and summarization. Specifically, all data were processed using the CONN toolbox (version 22.a; Gabrieli Lab, MIT McGovern Institute for Brain Research, Cambridge, MA, USA; https://web.conn-toolbox.org) implemented in MATLAB R2024a (MathWorks, Natick, MA, USA). To minimize inter-individual variability and fluctuations in functional connectivity (FC), denoising procedures were applied using CONN’s default preprocessing pipeline, which includes anatomical component-based noise correction (aCompCor). Confounding factors regressed out included signals from white matter, cerebrospinal fluid (CSF), realignment parameters, and scrubbing (outlier detection). Motion-related artifacts and physiological noise were also mitigated during this step. Subsequently, rs-fMRI data were temporally bandpass filtered at 0.008–0.09 Hz to reduce the influence of low-frequency scanner drift and high-frequency physiological noise such as respiratory and cardiac fluctuations.

***Preprocessing of MRI data***

Preprocessing of MRI data was conducted using the CONN toolbox. Functional images were first realigned to the anatomical reference and unwrapped to correct for geometric distortions. Slice-timing correction was applied, and outlier volumes were identified using scrubbing procedures based on the Artifact Detection Toolbox (ART). Both functional and structural images were normalized to the standard Montreal Neurological Institute (MNI) space and segmented into gray matter, white matter, and cerebrospinal fluid compartments. Functional data were then spatially smoothed with an 8-mm full-width at half-maximum (FWHM) Gaussian kernel. To ensure data quality and reliability, a series of quality control procedures were implemented. These included visual inspection for artifacts and anatomical abnormalities, as well as motion assessment using framewise displacement (FD). Scans with FD values exceeding 1.6 mm were excluded from further analysis.

***Selection of regions of interest (ROIs) & ROI-to-ROI analyses***

Given that sleep regulation involves a wide range of brain regions across cortical and subcortical structures, we aimed to adopt a comprehensive approach in selecting ROIs. To ensure sufficient anatomical coverage and standardization, we utilized the default atlas provided by the CONN functional connectivity toolbox, which is derived from the Harvard-Oxford cortical and subcortical structural atlases and the AAL cerebellar atlas (132 ROIs). This standardized parcellation enabled consistent ROI definition across participants and ensured the inclusion of brain areas potentially involved in sleep-related processes.

For the ROI-to-ROI statistical analyses, an analysis of covariance (ANCOVA) was conducted to compare FC between the active-cTBS and sham-cTBS groups at baseline and post-treatment stages, controlling for age, gender, and symptom duration as covariates. The analyses included 132 whole-brain ROIs, with statistical significance defined as p < 0.05, corrected for multiple comparisons using the false discovery rate (FDR) method across all ROI-to-ROI connections.

**7.8** **Subsequent assessments**

Insomnia severity and psychobehavioral assessments will be conducted after 2 weeks of treatment and at a 6-week follow-up, including PSQI, ESS, ISI, HAMA, and HAMD. We will record adverse events at each visit.

**7.9** **Flowchart of study assessments**

| Study Period | Baseline assessment | Intervention period | Post-intervention assessment | Follow-up assessment |
| --- | --- | --- | --- | --- |
| Weeks | Week 0 | Week 1-Week 2 | Week 2 | Week 6 |
| Demographics | × |  |  |  |
| Patient details | × |  |  |  |
| informed consent | × |  |  |  |
| Eligibility cheek and inclusion | × |  |  |  |
| PSQI | × |  | × | × |
| ESS | × |  | × | × |
| ISI | × |  | × | × |
| HAMA | × |  | × | × |
| HAMD | × |  | × | × |
| Brain MRI-T1 | × |  |  |  |
| Brain MRI-BOLD | × |  | × |  |

**7.10** **End of the trial and stopping rules**

**7.10.1** **Definition of end of the trial**

The end of the trial will be the last visit date for the last participant.

**7.10.2 Discontinuation or withdrawal of participants and 'stopping rules'**

Common adverse effects of cTBS include dizziness, headache, tinnitus, and a tingling or burning sensation on the scalp, which usually eases spontaneously without symptomatic treatment. Rare adverse reactions to cTBS include seizures, for which the physician will administer appropriate symptomatic treatment.

Patients who wish to discontinue participation in this trial are free to opt out. Reasons for withdrawal will be sought from all individuals and recorded. We will systematically record adverse events throughout the trial for all patients who wish to withdraw from the trial. Anyone who experiences an adverse event due to participating in the trial will be given appropriate medical advice and treatment.

**8** **Data management and quality assurance**

**8.1** **Confidentiality**

All the data will be handled using the MS Excel software. Detailed patient information is only available for this trial and not for any other purpose.

**8.2** **Data collection tools and source document identification**

The investigator will design and produce the case report form. The sponsor will approve the final version. All data will be entered clearly with a ballpoint pen in black/blue-black ink. If an error occurs, it will be crossed out with a line so the original entry can still be read. The correct entry will be inserted clearly, and the person who modified it will initialize and date it. No rewriting or use of correction fluid is allowed.

**9** **Record keeping and archiving**

The Principal Investigator (PI) is responsible for the secure archiving of trial files and databases. The field documents, CRFs, consent forms, and trial databases will be retained for 10 years after the study ends. If future data indicate this may be of interest or substantial, the data will be retained for additional retrospective subgroup analysis or long-term outcome follow-up.

**10** **Outcome indicators**

**10.1** **Primary endpoints**

The percentage of PSQI score improvement ≥3 points compared with baseline after the end of 10 treatments.

**10.2** **Secondary endpoints**

The secondary outcomes included the changes in scores on PSQI, ESS, ISI, HAMA, and HAMD from W0 to W2 and W6. The exploratory secondary outcomes are the changes in MRI from W0 to W2. MRI mainly analyzes brain functional connectivity.

**11** **Statistical analysis**

Primary analyses were performed in the intention-to-treat (ITT) population and repeated in the per-protocol (PP) population. In the primary ITT analysis, missing clinical data were imputed using last observation carried forward (LOCF), consistent with the original statistical analysis plan. Given the known limitations of LOCF, sensitivity analyses were additionally conducted using (1) linear mixed-effects models fitted to the raw observed data without imputation and (2) multiple imputation by chained equations (MICE) with predictive mean matching for continuous clinical outcomes. Twenty imputed datasets were generated, analyzed separately, and pooled according to Rubin’s rules. Consistency across these approaches was used to assess the robustness of the findings. Analyses of fMRI included only participants with complete imaging data, and safety analyses included all enrolled participants.

Continuous variables were summarized using descriptive statistics, including means and standard deviations (SDs), or medians and interquartile ranges (IQRs) or 95% confidence intervals (CIs), as appropriate. Categorical variables were presented as counts and percentages. Baseline characteristics were compared using the Student t-test, Wilcoxon rank-sum test, or Fisher’s exact test, as appropriate. The primary endpoint was analyzed using logistic regression adjusted for age, sex, disease duration, and baseline PSQI score. Secondary clinical outcomes were analyzed using linear mixed-effects models including post-baseline visits (weeks 2 and 6), with group, time, and group×time interaction as fixed effects, baseline value as a covariate, and participant as a random intercept. Adjusted between-group differences were estimated from marginal means with 95% CIs; degrees of freedom were computed using the Satterthwaite method, and Holm correction was applied for multiple testing. Prespecified models included a baseline-adjusted model and a fully adjusted model additionally controlling for age, sex, and disease duration. The fMRI ROI analyses used analysis of covariance with age, sex, and symptom duration as covariates; ROI-to-ROI multiple comparisons were controlled using false discovery rate correction. Two-sided P<0.05 was considered significant; for brain network analyses, significance was defined as FDR-adjusted P<0.05. Analyses were performed in SPSS 23, R 4.3.0, and DPABI v8.3 in MATLAB. Detailed methods are described in the Supplementary Materials.

**12 Ethics**

This clinical study will follow Helsinki's World Medical Assembly Declaration and other relevant regulations. The ethics committee will approve the protocol before the initiation of the study.

**13** **Organization**

**13.1** **Executive Committee (EC)**

Overall responsibility includes executing the study design, protocol, and publications. The members of EC are as follows:

Prof. Wen Jiang (Chair, PI), Department of Neurology, Xijing Hospital, Air Force Medical University, Xi'an, China

Prof. Feng Xia, Department of Neurology, Xijing Hospital, Air Force Medical University, Xi'an, China

Prof. Jingjing Zhao, Department of Neurology, Xijing Hospital, Air Force Medical University, Xi'an, China

Prof. Gengyao Hu, Department of Neurology, Xijing Hospital, Air Force Medical University, Xi'an, China

Dr. Xinyao Liu, Department of Neurology, Xijing Hospital, Air Force Medical University, Xi'an, China

Dr. Changgeng Song, Department of Neurology, Xijing Hospital, Air Force Medical University, Xi'an, China

Dr. Dong Dong, Department of Neurology, Xijing Hospital, Air Force Medical University, Xi'an, China

Dr. Yuxuan Ma, Department of Neurology, Xijing Hospital, Fourth Military Medical University, Xi'an, China

Dr. Dianwei Wu, Department of Neurology, Xijing Hospital, Fourth Military Medical University, Xi'an, China

Dr. Xuan Wang, Department of Neurology, Xijing Hospital, Fourth Military Medical University, Xi'an, China

Dr. Jianmin Hao, Department of Neurology, Xijing Hospital, Fourth Military Medical University, Xi'an, China

Dr. Hui Li, Department of Neurology, Xijing Hospital, Fourth Military Medical University, Xi'an, China

**13.2** **Quality Control and Assurance Committee (QCAC)**

Overall responsibility includes monitoring the execution of the study, regular quality inspection, and monitoring blinded response variables. When early dramatic benefits or potentially harmful effects have been shown, the QCAC will report to the EC on recommendations to continue or temporarily halt recruitment to the study. The QCAC members are not directly involved in the trial. The members of QCAC are as follows:

Ms. Yuan Che, Department of Neurology, Xijing Hospital, Fourth Military Medical University, Xi'an, China

Ms. Dan Mi, Department of Neurology, Xijing Hospital, Fourth Military Medical University, Xi'an, China

Prof. Fang Yang, Department of Neurology, Xijing Hospital, Fourth Military Medical University, Xi'an, China

**13.3** **Statistical Analysis Center**

Overall responsibility is to make a statistical analysis plan and conduct statistical analysis. The members of the Statistical Analysis Center are as follows:

Dr. Dianwei Wu, Department of Neurology, Xijing Hospital, Fourth Military Medical University, Xi'an, China

Dr. Dong Dong, Department of Neurology, Xijing Hospital, Air Force Medical University, Xi'an, China

**14** **Publication policy**

The results of this trial will be submitted for publication in a peer-reviewed journal, in addition to reports at appropriate specialist conferences.

**15** **Statement of compliance**

The trial will be conducted in compliance with the protocol, GCP, and the applicable regulatory requirement(s).

**16** **References**

1. Xue T, Wu X, Chen S*, et al.* The efficacy and safety of dual orexin receptor antagonists in primary insomnia: A systematic review and network meta-analysis. Sleep Med Rev 2022, 61: 101573.

2. Riemann D, Nissen C, Palagini L, Otte A, Perlis ML, Spiegelhalder K. The neurobiology, investigation, and treatment of chronic insomnia. Lancet Neurol 2015, 14: 547-558.

3. George MS, Aston-Jones G. Noninvasive techniques for probing neurocircuitry and treating illness: vagus nerve stimulation (VNS), transcranial magnetic stimulation (TMS) and transcranial direct current stimulation (tDCS). Neuropsychopharmacology 2010, 35: 301-316.

4. Barker AT, Jalinous R, Freeston IL. Non-invasive magnetic stimulation of human motor cortex. Lancet 1985, 1: 1106-1107.

5. Nardone R, Sebastianelli L, Versace V*, et al.* Effects of repetitive transcranial magnetic stimulation in subjects with sleep disorders. Sleep Med 2020, 71: 113-121.

6. Jiang CG, Zhang T, Yue FG, Yi ML, Gao D. Efficacy of repetitive transcranial magnetic stimulation in the treatment of patients with chronic primary insomnia. Cell Biochem Biophys 2013, 67: 169-173.

7. Herrero Babiloni A, Bellemare A, Beetz G*, et al.* The effects of non-invasive brain stimulation on sleep disturbances among different neurological and neuropsychiatric conditions: A systematic review. Sleep Med Rev 2021, 55: 101381.

8. Riemann D, Spiegelhalder K, Feige B*, et al.* The hyperarousal model of insomnia: a review of the concept and its evidence. Sleep Med Rev 2010, 14: 19-31.

9. Chung SW, Hill AT, Rogasch NC, Hoy KE, Fitzgerald PB. Use of theta-burst stimulation in changing excitability of motor cortex: A systematic review and meta-analysis. Neurosci Biobehav Rev 2016, 63: 43-64.

10. Cole EJ, Phillips AL, Bentzley BS*, et al.* Stanford Neuromodulation Therapy (SNT): A Double-Blind Randomized Controlled Trial. Am J Psychiatry 2022, 179: 132-141.

11. Plante DT. The Evolving Nexus of Sleep and Depression. Am J Psychiatry 2021, 178: 896-902.

12. Pang R, Zhan Y, Zhang Y*, et al.* Aberrant Functional Connectivity Architecture in Participants with Chronic Insomnia Disorder Accompanying Cognitive Dysfunction: A Whole-Brain, Data-Driven Analysis. Front Neurosci 2017, 11: 259.

13. Tahmasian M, Noori K, Samea F*, et al.* A lack of consistent brain alterations in insomnia disorder: An activation likelihood estimation meta-analysis. Sleep Med Rev 2018, 42: 111-118.

14. Krakauer JW, Shadmehr R. Consolidation of motor memory. Trends Neurosci 2006, 29: 58-64.

15. DelRosso LM, Hoque R. The cerebellum and sleep. Neurol Clin 2014, 32: 893-900.

16. Marchesi GF, Strata P. Climbing fibers of cat cerebellum: modulation of activity during sleep. Brain Res 1970, 17: 145-148.

17. Marchesi GF, Strata P. Mossy and climbing fiber activity during phasic and tonic phenomena of sleep. Pflugers Arch 1971, 323: 219-240.

18. De Zeeuw CI, Hoebeek FE, Schonewille M. Causes and consequences of oscillations in the cerebellar cortex. Neuron 2008, 58: 655-658.

19. Popa D, Spolidoro M, Proville RD, Guyon N, Belliveau L, Lena C. Functional role of the cerebellum in gamma-band synchronization of the sensory and motor cortices. J Neurosci 2013, 33: 6552-6556.

20. Niedermeyer E, Uematsu S. Electroencephalographic recordings from deep cerebellar structures in patients with uncontrolled epileptic seizures. Electroencephalogr Clin Neurophysiol 1974, 37: 355-365.

21. Huang Z, Li Y, Bianchi MT*, et al.* Repetitive transcranial magnetic stimulation of the right parietal cortex for comorbid generalized anxiety disorder and insomnia: A randomized, double-blind, sham-controlled pilot study. Brain Stimul 2018, 11: 1103-1109.

**Pittsburgh Sleep Quality** **Index (PSQI)**

INSTRUCTIONS: The following questions relate to your usual sleep habits during the past month only. Your answers should indicate the most accurate reply for the majority of days and nights in the past month. Please answer all questions.

1. During the past month, what time have you usually gone to bed at night?

BED TIME_______

2. During the past month, how long (in minutes) has it usually taken you to fall asleep each night?

NUMBER OF MINUTES_______

3. During the past month, what time have you usually gotten up in the morning?

GETTING UP TIME_______

4. During the past month, how many hours of actual sleep did you get at night?

HOURS OF SLEEP PER NIGHT_______

For each of the remaining questions, check the one best response. Please answer all questions.

5. During the past month, how often have you had trouble sleeping because you…
a) Cannot get to sleep within 30 minutes

Not during the past month_______	Less than once a week_______

Once or twice a week_______	Three or more times a week_______

b) Wake up in the middle of the night or early morning

Not during the past month_______	Less than once a week_______

Once or twice a week_______	Three or more times a week_______

c) Have to get up to use the bathroom

Not during the past month_______	Less than once a week_______

Once or twice a week_______	Three or more times a week_______

d) Cannot breathe comfortably

Not during the past month_______	Less than once a week_______

Once or twice a week_______	Three or more times a week_______

e) Cough or snore loudly

Not during the past month_______	Less than once a week_______

Once or twice a week_______	Three or more times a week_______

f) Feel too cold

Not during the past month_______	Less than once a week_______

Once or twice a week_______	Three or more times a week_______

g) Feel too hot

Not during the past month_______	Less than once a week_______

Once or twice a week_______	Three or more times a week_______

h) Had bad dreams

Not during the past month_______	Less than once a week_______

Once or twice a week_______	Three or more times a week_______

i) Have pain

Not during the past month_______	Less than once a week_______

Once or twice a week_______	Three or more times a week_______

j) Other reason(s), please describe____________________________

How often during the past month have you had trouble sleeping because of this?

Not during the past month_______	Less than once a week_______

Once or twice a week_______	Three or more times a week_______

6. During the past month, how would you rate your sleep quality overall?

Very good_______	Fairly good_______

Fairly bad_______	Very bad_______

7. During the past month, how often have you taken medicine to help you sleep?

Not during the past month_______	Less than once a week_______

Once or twice a week_______	Three or more times a week_______

8. During the past month, how often have you had trouble staying awake while driving, eating meals, or engaging in social activity?

Not during the past month_______	Less than once a week_______

Once or twice a week_______	Three or more times a week_______

9. During the past month, how much of a problem has it been for you to keep up enough enthusiasm to get things done?

Not during the past month_______	Less than once a week_______

Once or twice a week_______	Three or more times a week_______

**Insomnia Severity Index (ISI)**

1. Please rate the current (i.e., last 2 weeks) severity of your insomnia problems.

None	Mild	Moderate	Severe	Very

Difficulty falling asleep: 0	1	2	3	4

Difficulty staying asleep: 0	1	2	3	4

Problem waking up too early: 0	1	2	3	4

2. How satisfied/dissatisfied are you with your current sleep pattern?

Very Satisfied	Very Dissatisfied

0	1	2	3	4

3. To what extent do you consider your sleep problem to interfere with your daily functioning (e.g. daytime fatigue, ability to function at work/daily chores, concentration, memory, mood, etc.)

Not at all interfering	A little	Somewhat	Much	Very much interfering

0	1	2	3	4

4. How noticeable to others do you think your sleeping problem is in terms of impairing the quality of your life?

Not at all noticeable	Barely	Somewhat	Much	Very much noticeable

0	1	2	3	4

5. How worried/distressed are you about your current sleep problem?

Not at all	A little	Somewhat	Much	Very much

0	1	2	3	4

**The Epworth Sleepiness Scale (ESS)**

| How Sleepy Are You?  How likely are you to doze off or fall asleep in the following situations? You should rate your chances of dozing off, not just feeling tired. Even if you have not done some of these things recently try to determine how they would have affected you. For each situation, decide whether or not you would have:  · No chance of dozing =0  · Slight chance of dozing =1  · Moderate chance of dozing =2  · High chance of dozing =3  Write down the number corresponding to your choice in the right-hand column. Total your score below. | |
| --- | --- |
| **Situation** | **Chance of Dozing** |
| Sitting and reading |  |
| Watching TV |  |
| Sitting inactive in a public place (e.g., a theater or a meeting) |  |
| As a passenger in a car for an hour without a break |  |
| Lying down to rest in the afternoon when circumstances permit |  |
| Sitting and talking to someone |  |
| Sitting quietly after a lunch without alcohol |  |
| In a car, while stopped for a few minutes in traffic |  |

**Hamilton Anxiety Rating Scale (HAMA)**

|  | Not present | Mild | Moderate | Severe | Very severe |
| --- | --- | --- | --- | --- | --- |
| **1 Anxious mood:** Worries, anticipation of the worst, fearful anticipation, irritability**.** |  |  |  |  |  |
| **2 Tension:** Feelings of tension, fatigability, startle response, moved to tears easily, trembling, feelings of restlessness, inability to relax. |  |  |  |  |  |
| **3 Fears:** Of dark, of strangers, of being left alone, of animals, of traffic, of crowds. |  |  |  |  |  |
| **4 Insomnia:** Difficulty in falling asleep, broken sleep, unsatisfying sleep and fatigue on waking, dreams, nightmares, night terrors. |  |  |  |  |  |
| **5 Intellectual:** Difficulty in concentration, poor memory. |  |  |  |  |  |
| **6 Depressed mood:** Loss of interest, lack of pleasure in hobbies, depression, early waking, diurnal swing. |  |  |  |  |  |
| **7 Somatic (muscular):** Pains and aches, twitching, stiffness, myoclonic jerks, grinding of teeth, unsteady voice, increased muscular tone. |  |  |  |  |  |
| **8 Somatic (sensory):** Tinnitus, blurring of vision, hot and cold flushes, feelings of weakness, pricking sensation. |  |  |  |  |  |
| **9 Cardiovascular symptoms:** Tachycardia, palpitations, pain in chest, throbbing of vessels, fainting feelings, missing beat. |  |  |  |  |  |
| **10 Respiratory symptoms:** Pressure or constriction in chest, choking feelings, sighing, dyspnea. |  |  |  |  |  |
| **11 Gastrointestinal symptoms:** Difficulty in swallowing, wind abdominal pain, burning sensations, abdominal fullness, nausea, vomiting, borborygmi, looseness of bowels, loss of weight, constipation. |  |  |  |  |  |
| **12 Genitourinary symptoms:** Frequency of micturition, urgency of micturition, amenorrhea, menorrhagia, development of frigidity, premature ejaculation, loss of libido, impotence. |  |  |  |  |  |
| **13 Autonomic symptoms:** Dry mouth, flushing, pallor, tendency to sweat, giddiness, tension headache, raising of hair. |  |  |  |  |  |
| **14 Behavior at interview:** Fidgeting, restlessness or pacing, tremor of hands, furrowed brow, strained face, sighing or rapid respiration, facial pallor, swallowing, etc. |  |  |  |  |  |

**Hamilton Depression Rating Scale (HAMD)**

Classification of symptoms which may be difficult to obtain can be scored as:

0 - absent: 1 - doubtful or trivial: 2 - present.

Classification of symptoms where more detail can be obtained can be expanded to:

0 - absent; 1 - mild; 2 - moderate; 3 - severe; 4 - incapacitating.

| **HAMD Rating Scale Symptoms** | **score** |
| --- | --- |
| Depressed mood | 0 1 2 3 4 |
| Guilt feelings | 0 1 2 3 4 |
| Suicide | 0 1 2 3 4 |
| Insomnia - early | 0 1 2 |
| Insomnia - middle | 0 1 2 |
| Insomnia - late | 0 1 2 |
| Work and activities | 0 1 2 3 4 |
| Retardation - psychomotor | 0 1 2 3 4 |
| Agitation | 0 1 2 3 4 |
| Anxiety - psychological | 0 1 2 3 4 |
| Anxiety - somatic | 0 1 2 3 4 |
| Somatic symptoms GI | 0 1 2 |
| Somatic symptoms - General | 0 1 2 |
| Sexual dysfunction – menstrual disturbance | 0 1 2 |
| Hypochondria | 0 1 2 3 4 |
| Weight loss by history | 0 1 2 |
| Insight | 0 1 2 |
| -day and night | 0 1 2 |
| -change | 0 1 2 |
| Disintegration of personality or reality | 0 1 2 3 4 |
| Paranoia | 0 1 2 3 4 |
| Coercion | 0 1 2 |
| Sense of diminished capacity | 0 1 2 3 4 |
| Sense of despair | 0 1 2 3 4 |
| Sense of inferiority | 0 1 2 3 4 |

**Cerebellar continuous theta burst stimulation for insomnia disorder: a randomized sham-controlled trial**

**Statistical Analysis Plan (version 1.0)**

Research site: Xijing Hospital

Principle Investigator: Wen Jiang

Responsible department: Department of Neurology

Contact: +86-29-84771319

1. **Introduction**

This statistical analysis plan (SAP) details the planned statistical analyses for the " Cerebellar continuous theta burst stimulation for insomnia disorder: a randomized sham-controlled trial" and is based on the protocol, as well as any subsequent amendments. The SAP should be read together with the protocol.

2. **Study objective(s) and endpoint(s)**

**2.1 Study objective(s)**

**2.1.1 Primary objective**

To explore the efficacy and safety of continuous theta burst stimulation (cTBS) on the cerebellar dentate nucleus in treating insomnia disorder (ID).

**2.1.2 Secondary objective**

To evaluate the efficacy of CRB-cTBS in the treatment of ID, we will observe the changes in scores on the Pittsburgh Sleep Quality Index (PSQI), Epworth Sleepiness Scale (ESS), insomnia Severity Index (ISI), Hamilton Depression Scale (HAMD), and Hamilton Anxiety Scale (HAMA) after 10 sessions of CRB-cTBS treatment and at 6-week follow-up. The changes in the MRI was investigated.

**2.2** **Study endpoint(s)**

**2.2.1 Primary outcomes**

The percentage of PSQI score improvement ≥3 points compared with baseline after the end of 10 treatments.

2.2.2 **Secondary outcomes**

PSQI, ESS, ISI, HAMA, and HAMD scores change from baseline to 2-week and 6-week in the Active-cTBS and Sham-cTBS groups.

Changes of fMRI from baseline to 2-week in the Active-cTBS and Sham-cTBS groups.

2.2.3 **Safety outcomes**

Safety outcomes included the incidence of adverse events (AEs) and serious adverse events (SAEs) throughout the study. The common AEs of cTBS include dizziness, headache, tinnitus, and scalp tingling or burning, which usually resolve spontaneously without symptomatic treatment. Rare SAEs of cTBS include seizures, and appropriate symptomatic treatment will be given by the physician.

3. **Study design**

This study will be a randomized, parallel-controlled clinical study. Patients with ID who meet the inclusion and exclusion criteria will provide written informed consent. Patients will be randomly divided into the Active-cTBS and Sham-cTBS groups in a 1:1 ratio. They will be treated 5 times a week for 2 weeks, 10 times in total, and followed up for 4 weeks after treatment.

4. **Analysis populations**

Participants in our study were analyzed according to the intention-to-treat (ITT) principle. The analysis for the per-protocol (PP) population was repeated to assess the robustness of our conclusion.

5. **Missing data**

Every effort should be made to complete follow-up, primarily to assess primary efficacy outcomes. Missing outcome data will be verified at the last follow-up. In the primary ITT analysis, missing clinical data were imputed using last observation carried forward (LOCF), consistent with the original statistical analysis plan. Given the known limitations of LOCF, sensitivity analyses were additionally conducted using (1) linear mixed-effects models fitted to the raw observed data without imputation and (2) multiple imputation by chained equations (MICE) with predictive mean matching for continuous clinical outcomes. Twenty imputed datasets were generated, analyzed separately, and pooled according to Rubin’s rules. Consistency across these approaches was used to assess the robustness of the findings. Analyses of fMRI included only participants with complete imaging data, and safety analyses included all enrolled participants.

6. **Efficacy analysis**

Patients were analyzed according to the ITT principle. Continuous variables will be presented as descriptive statistics (mean with standard deviation [SD] or median with interquartile range [IQR]). Categorical variables will be summarized as counts and percentages. As appropriate, baseline characteristics will be compared between the groups using the Student's t-test, Wilcoxon test, or Fisher's exact test.

**6.1 Clinical data**

The primary endpoint was analyzed using logistic regression adjusted for age, sex, disease duration, and baseline PSQI score. Secondary clinical outcomes were analyzed using linear mixed-effects models including post-baseline visits (weeks 2 and 6), with group, time, and group×time interaction as fixed effects, baseline value as a covariate, and participant as a random intercept. Adjusted between-group differences were estimated from marginal means with 95% CIs; degrees of freedom were computed using the Satterthwaite method, and Holm correction was applied for multiple testing. Prespecified models included a baseline-adjusted model and a fully adjusted model additionally controlling for age, sex, and disease duration.

**6.2 MRI data**

For the fMRI of regions of interest (ROI)-to-ROI statistical analyses, analysis of covariance was conducted to compare functional connectivity (FC) between pre- and post-treatment conditions in patients, with age, gender, and symptom duration included as covariates. The analysis was performed across 132 whole-brain ROIs, corrected for multiple comparisons using the false discovery rate (FDR) across all ROI-to-ROI connections. Two-sided P values <0.05 were considered statistically significant. Analyses were performed using SPSS (version 23), R (version 4.3.0), and the DPABI toolbox (v8.3) in MATLAB.

**7. Safety analysis**

All the safety events will be recorded. Wilcoxon rank sum test and chi-square test will be used for data analysis. *P* < 0.05 will be considered statistically significant. SPSS 23 was used for statistical analysis.

**Informed Consent Form**

**Information Page**

Dear participants:

We invite you to participate in a randomized, controlled clinical study targeting the cerebellum with continuous theta burst stimulation (cTBS) patterned transcranial magnetic stimulation (TMS) to treat insomnia disorder. This study aims to evaluate its efficacy and safety, provide new therapeutic approaches for insomnia disorder, and improve patients' quality of life. This research protocol has been reviewed and approved by the Ethics Committee of the First Affiliated Hospital of Air Force Medical University (Xijing Hospital) for clinical research.

Before you decide whether to participate in this study, please read the following information carefully. It will help you understand the study's purpose, procedures, duration, potential benefits, risks, and discomforts. If you wish, you can also discuss it with your family, friends, or doctor to help you make a decision.

1. Research Background and Objectives

1.1 Research Background

Insomnia disorder is a significant global public health issue, with nearly one-third of the general population experiencing symptoms of insomnia during their lifetime. The main clinical manifestations include difficulty falling asleep (sleep latency exceeding 30 minutes), sleep maintenance problems (awakening ≥2 times per night), early morning awakening, reduced sleep quality, and shortened total sleep time (typically less than 6.5 hours), often accompanied by daytime functional impairments. Daytime dysfunction resulting from insomnia commonly includes fatigue, low mood, physical discomfort, and cognitive impairment. Repetitive transcranial magnetic stimulation (rTMS) utilizes time-varying magnetic fields to induce electrical currents, delivering pulsed magnetic fields to the cerebral cortex, thereby depolarizing cortical neurons, triggering action potentials, and modulating cortical electrical activity. Patterned rTMS is an advanced stimulation protocol; a commonly used form is TBS, characterized by burst stimulation with each burst usually containing three pulses, an intraburst frequency of 50 Hz, and an interburst frequency of 5 Hz. Continuous TBS predominantly exerts inhibitory effects on the cortex. rTMS is non-invasive, safe, and repeatable. Stimulation of cerebellar Purkinje inhibitory fiber projections can further reduce cortical excitability.

1.2 Research Objectives

Therefore, this study aims, for the first time, to apply repetitive transcranial magnetic stimulation using a cTBS protocol targeted at the cerebellum for the treatment of insomnia disorder, utilizing neuronavigation in combination with magnetic resonance imaging (MRI) for precise localization. The objective is to further enhance the efficacy and durability of rTMS treatment by improving and optimizing rTMS and related techniques. This study will be conducted in the Department of Neurology at Xijing Hospital, with an anticipated enrollment of 68 voluntary participants. The Ethics Committee of Xijing Hospital has reviewed and confirmed that this study complies with the principles of the Declaration of Helsinki and meets medical ethical standards.

2. Who Should Not Participate in This Study

According to the objectives of this study, the following individuals are not suitable for participation: 1) Those who are unable to cooperate with the treatment and required assessments; 2) Individuals with other types of sleep disorders (such as sleep-related movement disorders, hypersomnia or parasomnias, sleep apnea-hypopnea syndrome, etc.); 3) Those with a history of psychiatric medication or alcohol dependence or abuse within the past two years; 4) Individuals who have traveled or plan to travel across time zones (≥3 time zones) within one month prior to the trial or during the study period; 5) Individuals whose work involves night shifts or shift work; 6) Those who plan to undergo surgical treatment within one month before or during the study; 7) Individuals with comorbidities such as thyroid dysfunction, Parkinson's syndrome, severe infections, cerebrovascular diseases, malignant tumors, significant dysfunction of the heart, liver, or kidneys, or those with psychiatric disorders; 8) Individuals with a history of epilepsy; 9) Pregnant or breastfeeding women; 10) Individuals with contraindications to TMS or MRI, such as having metal implants or implanted devices (e.g., pacemakers, deep brain stimulators, etc.).

3. What Will Be Required If You Participate in the Study?

3.1 Pre-enrollment Assessments

Before enrolling, the physician will collect and record your medical history, complete relevant clinical rating scales, and perform necessary auxiliary examinations, such as cranial MRI. If you meet the eligibility criteria, you may voluntarily participate in the study and sign the informed consent form. If you choose not to participate, your decision will be fully respected.

3.2 Study Procedures

If you agree to participate, the study will proceed as follows:

(1) Collection of your relevant clinical data and results of auxiliary examinations;

(2) A two-week course of treatment;

(3) Follow-up one month after completion of the treatment.

3.3 Additional Requirements

You must accurately report your clinical information to the study team, which is crucial for evaluating treatment outcomes. You must also comply with follow-up requirements, as these are essential for assessing the effectiveness of the treatment.

4. Potential Benefits of Participation

4.1. Benefits to the Individual Participant

You may benefit from participating in this study. Such benefits could include improvement in your insomnia disorder and enhancement of your quality of life. You will also receive quality medical care during the study period.

4.2. Benefits to Society

The broader community may benefit from this research. These benefits include the potential for improved sleep and quality of life for patients with insomnia disorder, the promotion of new treatment approaches, and reduced social burden. The findings may also inform more scientific treatment options for you and other patients with similar conditions in the future.

5. Possible Adverse Reactions, Risks, and Discomforts

The risks of TMS are low and may include scalp tingling or burning sensations and, rarely, seizures. Scalp discomfort is more common and generally resolves on its own. Seizures are rare during treatment; if one occurs, treatment will be stopped immediately, and you will receive appropriate medical care and compensation per applicable laws and regulations.

6. Costs

The costs of TMS treatment, neuropsychological tests, and imaging examinations involved in this study will not be borne by you. No other compensation or payment will be provided for participation.

7. Confidentiality of Personal Information

Your medical records (study files, test results, etc.) will be securely stored at the hospital where you receive care. Physicians will record examination results in your medical file. Researchers, the ethics committee, and regulatory authorities may access your medical records for study purposes. Any published results from this research will not disclose your identity. Within the limits permitted by law, all efforts will be made to protect the privacy of your medical information. According to medical research ethics, study data may be made available for public inquiry and sharing except for personal privacy information, but only through web-based electronic databases, ensuring that no personal privacy information is disclosed.

8. How to Obtain More Information

You may ask the investigator any questions about this study at any time and will receive appropriate answers. Your physician will promptly inform you if any critical new information arises during the study that might affect your willingness to continue participating.

9. Voluntary Participation and Withdrawal

Participation in this study is entirely voluntary. You may refuse to participate or withdraw from the study at any time without affecting your relationship with your physician, your medical care, or your access to other benefits. For your best interest, the investigator or physician may also terminate your participation in the study at any time.

10. What Should You Do Now?

Whether or not to participate in this study is your decision (and that of your family). Before deciding, please ask your doctor any questions you may have. Thank you for reading this information. If you choose to participate, please inform your study investigator, and arrangements will be made for your participation. If you have any ethical concerns during the study, please get in touch with the Ethics Committee of Xijing Hospital. Please retain this information sheet for future reference.

**Informed Consent Form**

**Signature Page**

I have read the information about this study and have had the opportunity to discuss it with my doctor. All my questions have been answered satisfactorily.

I understand the potential risks and benefits of participating in this study. I confirm that my participation is voluntary and that:

●I have had sufficient time to consider my decision.

●I may withdraw from the study without any negative consequences to my medical care or rights.

If I withdraw from the study, I will inform my doctor of any changes in my condition and complete the necessary physical and laboratory examinations, which will benefit me and the study.

I agree that the Ethics Committee or the study sponsor's representatives may access my study records.

I agree ☐ / disagree ☐ to using my medical records and pathological samples for purposes other than this study.

I will receive a signed copy of this informed consent form with the date noted.

Finally, I agree to participate in this study and follow the doctor's instructions.

Participant's Signature: ______

Participant's Contact Number: ______

Date: ______

Doctor's Declaration

I confirm that I have fully explained the details of this study to the participant, including their rights, potential benefits, and risks. I have provided them with a signed copy of this informed consent form.

Researcher's Signature: ______

Date: ______
